# MO-MEMES: A method for accelerating virtual screening using multi-objective Bayesian optimization

**DOI:** 10.3389/fmed.2022.916481

**Published:** 2022-09-23

**Authors:** Sarvesh Mehta, Manan Goel, U. Deva Priyakumar

**Affiliations:** Center for Computational Natural Science and Bioinformatics, International Institute of Information Technology, Hyderabad, India

**Keywords:** drug discovery, machine learning, virtual screening, Bayesian optimization, chemical space exploration, High throughout screening

## Abstract

The pursuit of potential inhibitors for novel targets has become a very important problem especially over the last 2 years with the world in the midst of the COVID-19 pandemic. This entails performing high throughput screening exercises on drug libraries to identify potential “hits”. These hits are identified using analysis of their physical properties like binding affinity to the target receptor, octanol-water partition coefficient (LogP) and more. However, drug libraries can be extremely large and it is infeasible to calculate and analyze the physical properties for each of those molecules within acceptable time and moreover, each molecule must possess a multitude of properties apart from just the binding affinity. To address this problem, in this study, we propose an extension to the Machine learning framework for Enhanced MolEcular Screening (MEMES) framework for multi-objective Bayesian optimization. This approach is capable of identifying over 90% of the most desirable molecules with respect to all required properties while explicitly calculating the values of each of those properties on only 6% of the entire drug library. This framework would provide an immense boost in identifying potential hits that possess all properties required for a drug molecules.

## 1. Introduction

Drug discovery is a long, expensive, and extremely laborious process that involves multiple steps with knowledge from a wide variety of domains like chemistry, biology and pharmacology. The first step in this process is the identification of potential hit molecules for a novel target followed by experimental evaluation typically using biochemical assays toward lead identification. These hits are then optimized to have higher binding affinity, low toxicity, and improved bioavailability among other requirements. The time and expense involved in this process has given rise to alternate *in silico* approaches like virtual screening wherein molecules are computationally evaluated to identify potential hits. The structure based drug design (SBDD) method, docking, is used most commonly in virtual screening to identify molecules with high binding affinity to the given target (1–4).

The availability of large scale open source datasets in molecular sciences has opened up the avenue for the application of a wide array of modern machine learning methods in this domain (5, 6). This includes problems like physical property prediction (7–9), drug design (10), protein structure predictions (11, 12), molecular simulations (13–16) and *de novo* molecule generation (17). Most *de novo* molecule generation approaches are based on recurrent neural networks (18, 19), variational autoencoders (20–22), generative adverserial networks (23–26), reinforcement learning (27–30). These methodologies have shown great promise in molecule generation with desirable properties like quantitative estimate of drug likeliness (QED), octanol parition coefficient (LogP) and docking scores but Gao and Coley found that a large number of the generated molecules though novel and diverse, are infeasible to synthesize (31).

In comparison, molecules present in drug libraries enumerated through simple reactions can also be novel, diverse and synthesizable with a probability of ≈86% (31, 32). However, virtual screening of large molecule libraries can be extremely time consuming since finding the most stable protein-ligand conformation is a non-convex optimization problem making each docking calculation extremely slow. Even in the most comprehensive study by (33) approximately 10^8^ molecules were docked, but that is still a very small number in comparison to the vast ZINC20 library with about 1.4 billion molecules (34). Moreover, their study also showed that hits for a target can be identified using only the top fraction of the ligands with respect to the docking score. This posits the argument for efficiently sampling from the chemical space to find molecules with high docking scores.

The DeepDock algorithm by Liao et al. helped in this regard by augmenting the SBDD process and managed to obtain top 60% of the high scoring molecules with 50 times fewer docking calculations and Graff et al. proposed the application of pool based active learning for identifying potential hits (35, 36). Gupta and Zhou clustered the molecules based on molecular properties and performed limited docking to improve high throughput virtual screening (37). We proposed MEMES which uses Bayesian optimization on the chemical space to find the top scoring molecules and using gaussian process regression to estimate the protein-ligand docking score. We showed that the proposed framework was able to identify most of the top scoring molecules by performing docking calculations on only 6% of the molecules in the drug library and showed its application on multiple drug libraries and proteins. However, finding molecules with the highest docking scores is not enough since drug molecules must also possess other properties like high QED and LogP between 1 and 5 and it was found that most high scoring drug molecules violate one or more of these constraints. Hence, there is a requirement for frameworks that can optimize for multiple properties during high throughput virtual screening like the work by Baird et al. (38).

**Figure 1:**
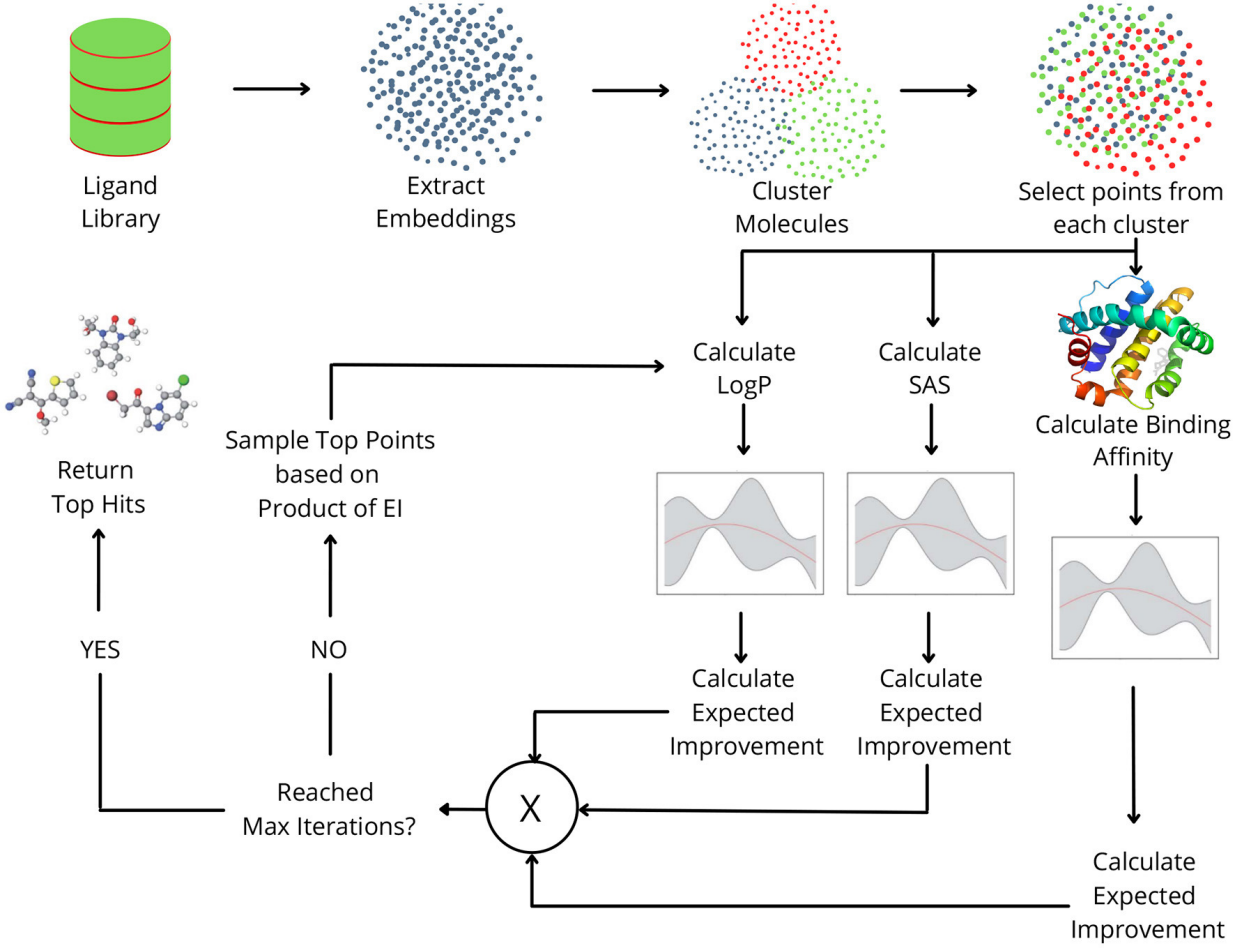
Overview of the MO-MEMES pipeline. Embeddings for small drug-like molecules in ligand library are extracted and clustered. Points are sampled randomly from each cluster and respective properties are calculated for each of them and then used to train gaussian process regression.

In this study, we propose *MO-MEMES* (Figure 1), a machine learning based framework for finding the top hits in a drug library with respect to multiple properties simultaneously. To achieve this, we perform multi-objective Bayesian optimization to find molecules that lie at the pareto front with respect to the required properties. A small subset of the library is sampled initially and all the properties are calculated for them. This training set is iteratively augmented using an acquisition function which aims to find molecules that show an improvement for as many properties as possible. We experiment with two acquisition functions and show their application on different combinations of properties. This methodology was successful in finding a large number of molecules at/near the pareto front while performing docking calculations on only 6% of the ligands.

## 2. Theory and methods

For the purpose of this study, we have extended the MEMES framework by Mehta et al. (39) due to its excellent performance using new acquisition functions to account for multiple properties during Bayesian optimization (40, 41). This section describes the docking methodology, molecular representation, an overview of MEMES and the acquisition function.

### 2.1. Docking methodology

Molecular docking is a powerful tool for measuring the binding affinity of a ligand with a protein receptor using a simple scoring function. Hence, it is extremely useful in identifying potential inhibitors from small molecule libraries. Ligand and receptor preparation were done using AutoDock 4.2 (42). For the purpose of this study, the ZINC-250K dataset, a subset of the ZINC15 (43) database of drug like molecules was used to identify the top hits for inhibiting the Tau Tubulin Kinase 1 and SARS CoV-2 *M*_*pro*_ proteins.

### 2.2. Machine learning framework for Enhanced MolEcular Screening (MEMES)

The MEMES framework uses Bayesian optimization to find the potential inhibitors for a target from the given drug library. Bayesian optimization is especially useful for optimizing expensive black-box function like binding affinity. There are two main components in Bayesian optimization: a surrogate function that can be used to approximate the black box and an acquisition function to determine the next points to sample. In this work we have used Gaussian Process Regression (ExactGP) and Deep Gaussian Processes (DeepGP) as the surrogate functions along with two types of molecular descriptors: mol2vec and CDDD (44, 45). The details regarding the ExactGP are provided in Section 2.2.1, DeepGP in the Supplementary material and acquisition function in Section 2.2.2.

#### 2.2.1. Gaussian process regression (GPR)

Gaussian process regression is a non-parametric Bayesian regression technique. In Bayesian statistics it assumed that all the *k* points in the initial dataset are drawn at random from a prior multivariate gaussian distribution given by:


(1) $$\begin{array}{l} f\left(x_{1:k}\right)\sim \text{N}\left(\mu_{0}\left(x_{1:k}\right),\Sigma_{0}\left(x_{1:k},x_{1:k}\right)\right) \end{array}$$

The mean vector is obtained by the evaluation of the mean function (μ_0_) at each data point and the covariance matrix is obtained by the evaluation of the covariance function or kernel (Σ_0_) at each pair of points. The choice of the kernel function must be such that a strong correlation exists between points and closer to each other and the resulting covariance matrix be positive semi definite. Suppose the prior distribution is constructed for *n* points. For a point *x* at *k* = *n* + 1, the distribution is obtained from Baye's rule:


(2) $$\begin{array}{l} f\left(x\right)|f\left(x_{1:k}\right)\sim \text{N}\left(\mu_{n}\left(x\right),\sigma_{n}^{2}\left(x\right)\right) \end{array}$$

(3) $$\begin{array}{l} \mu_{n}\left(x\right)=\Sigma_{0}\left(x,x_{1:k}\right)\Sigma_{0}{\left(x,x_{1:k}\right)}^{-1}\left(f\left(x_{1:n}\right)-\mu_{0}\left(x_{1:n}\right)\right)+\mu_{0}\left(x\right) \end{array}$$

(4) $$\begin{array}{l} \sigma_{n}^{2}\left(x\right)=\Sigma_{0}\left(x,x\right)-\Sigma_{0}\left(x,x_{1:n}\right)\Sigma_{0}{\left(x_{1:n},x_{1:n}\right)}^{-1}\Sigma \left(x_{1:n},x\right) \end{array}$$

The conditional probability distribution is called the posterior probability distribution. For faster computations, the matrix inversions are obtained through Cholesky decompositions and solving a system of linear equations. The implementation of exact gaussian processes in GPyTorch is used in this work (46).

#### 2.2.2. Expected improvement (EI)

The acquisition function is used to find points to be added to the dataset during Bayesian optimization. For maximizing the black box function, the new points must possess a balance between exploring unknown regions of the space as well as exploiting the information about where the function value is maximum. The acquisition function is responsible for finding such points and Expected Improvement is one such function. The improvement (*I*) at a point *x* is defined as


(5) $$\begin{array}{l} I=max\left(0,f\left(x\right)-f^{*}\right) \end{array}$$

In Equation (5), f^*^ refers to best function value found so far. In this scenario, since gaussian processes are being used, *f*(*x*) is a random value ~*N*(μ, σ^2^) where μ and σ correspond to a mean and variance evaluated at point *x*. The expected improvement is then defined as


(6) $$\begin{array}{l} EI\left(x\right)=𝔼\left[max\left(0,f\left(x\right)-f^{*}\right)\right] \end{array}$$

After integrating the reparameterized distribution (*x* = μ + σϵ) (47), the obtained expected improvement for a point *x* is given by


(7) $$\begin{array}{l} EI\left(x\right)=\sigma \left(x\right)Z\Phi \left(Z\right)+\sigma \left(x\right)\phi \left(Z\right) \end{array}$$

where


(8) $$\begin{array}{l} Z=\frac{\mu \left(x\right)-f^{*}-\zeta }{\sigma \left(x\right)} \end{array}$$

In Equation (7), Φ and ϕ are the cumulative distribution function and probability distribution function respectively. The term ζ determines the degree of exploration during optimization.

### 2.3. Multi objective Bayesian optimization

Multiobjective optimization is a significantly more complex problem than single objective optimization since in this case optimal decisions have to be taken considering the trade-offs between conflicting objectives. The task is to find the pareto frontier which is the set of points such that no objective can be improved without making another objective worse. This problem becomes significantly harder when we work with black box functions in high dimensional space and hence, we try to extend the single objective Bayesian optimization approach mentioned in the previous sections to multiple objectives.

Multiobjective Bayesian optimization also consists of two parts: the surrogate model and the acquisition function. For the surrogate, we continue to use exact gaussian processes. However, the choice of acquisition functions is an active area of research since the acquisition function for multiple objective along with balancing exploration and exploitation must also promote improvement for as many objectives as possible in order to identify the pareto optimal points. These include work by Daulton et al. (40, 41) and Suzuki et al. (48).

#### 2.3.1. Acquisition function

For the purpose of this study, exact gaussian processes are trained on each of the given objectives separately and then used to calculate the expected improvement for each point in the dataset. The expected improvements from each objective are then multiplied and the product of individual expected improvements is then used as the acquisition function.


(9) $$\begin{array}{l} EI\left(x\right)=EI_{1}\left(x\right)\times EI_{2}\left(x\right).......\times EI_{n}\left(x\right) \end{array}$$

In the aforementioned equation, *EI*_*i*_(*x*) is the expected improvement of the *i*th objective. The molecules with the highest *EI*(*x*) are then inducted into the training set and the process is then repeated till a preset number of molecules is reached.

## 3. Results and discussion

In this section, experiments were performed on different combinations of properties and proteins to validate the performance of the proposed framework. The proteins used for this study are

- SARS CoV-2 *M*_*pro*_ (PDB ID: 6LU7): With the world in the midst of a global pandemic caused by COVID-19, the main protease (*M*_*pro*_) has been identified as an important target due its vital role in viral transcription and replication (49).
- Tau Tubulin Kinase 1 (PDB ID: 4BTK): Neurodegenerative diseases have become extremely common over the past few years, and the tau-tubulin kinase 1 has proved to be an attractive target to combat a wide variety of neurodegenerative diseases (50).

In order to model this as a multi-objective maximization problem, transformations are applied on all three properties. Binding affinity and Synthetic Accessibility Score (SAS) are multiplied by -1 since the values need to be as low as possible. A gaussian transformation is applied on the LogP values such that there is a peak at 2.5. The experiments performed in this study are listed in Table 1.

**Table 1:** List of experiments performed to validate the performance of MO-MEMES.

**Protein**	**Binding affinity**	**LogP**	**SAS**	**Descriptor**
4BTK	×	×		CDDD
4BTK	×		×	CDDD
4BTK		×	×	CDDD
6LU7	×	×		CDDD
6LU7	×		×	CDDD
6LU7		×	×	CDDD
4BTK	×	×		Mol2vec
4BTK	×		×	Mol2vec
4BTK		×	×	Mol2vec
6LU7	×	×		Mol2vec
6LU7	×		×	Mol2vec
6LU7		×	×	Mol2vec
6LU7	×	×	×	CDDD
4BTK	×	×	×	CDDD

### 3.1. Exact MO-MEMES

This section describes the results obtained by applying the Exact MO-MEMES architecture in screening the ZINC-250K library to find potential hits for 6LU7 and 4BTK. Section 3.1.1 elaborates the performance of MO-MEMES on different combinations of two properties and Section 3.1.2 talks about how MO-MEMES works on sampling molecules that possess three properties simultaneously.

#### 3.1.1. Optimizing two properties

**Figure 2:**
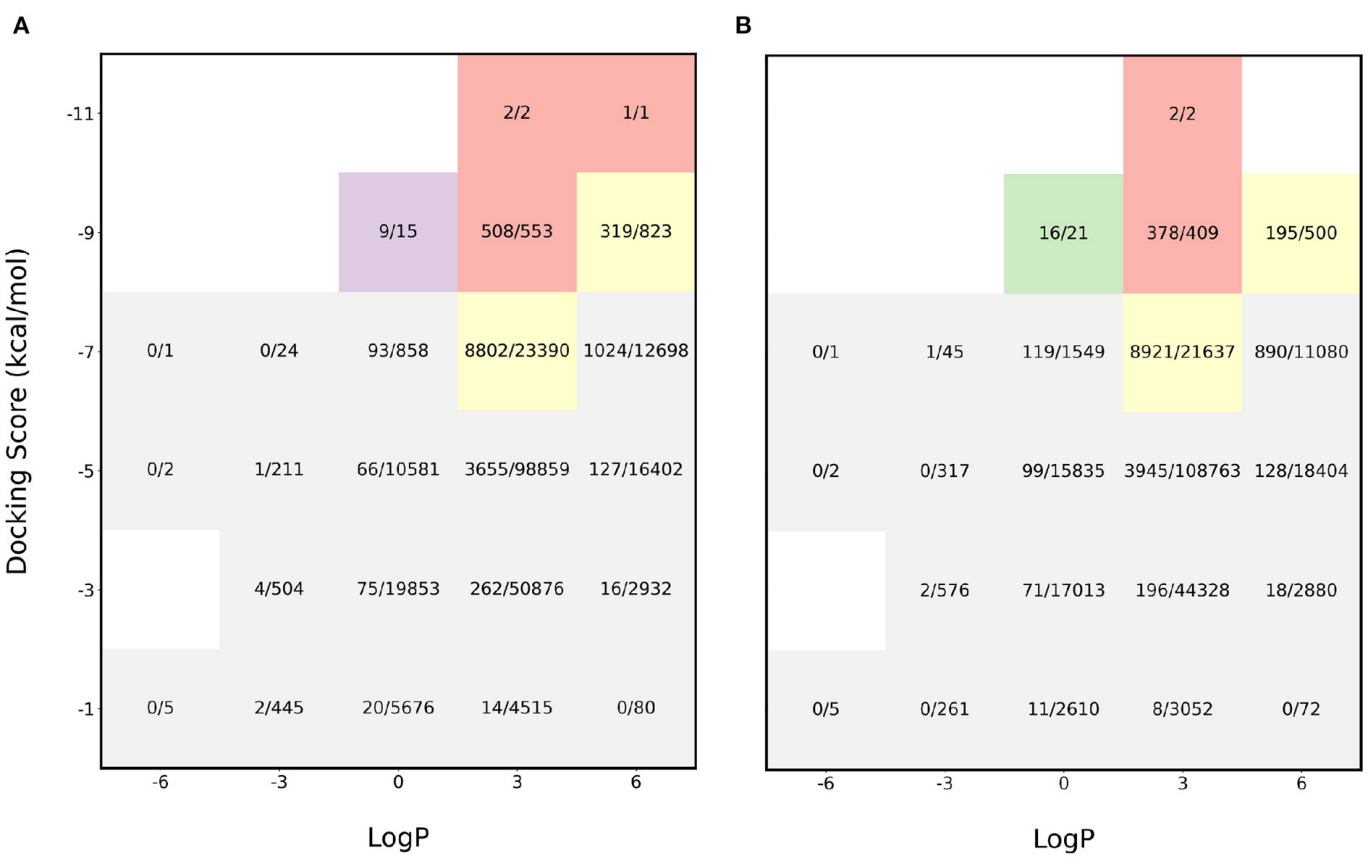
Heatmap of fraction of molecules sampled from each bucket of docking score and LogP. **(A)** shows the heatmap for protein target 4BTK while **(B)** shows for 6LU7.

The initial experiments were performed to see how the pipeline performs for sampling molecules with LogP close to 2.5 and a high binding affinity. We find that the proposed acquisition performs really well in identifying the molecules with desirable properties and this is shown in Figure 2. The red sections in the heatmap show that more than 90% of the desirable molecules were sampled i.e., molecules with binding affinity < −8 kcal/mol and LogP between 0 and 5 while performing docking calculations on only 6% of the entire dataset. Furthermore, the application of gaussian function on the LogP to identify molecules with LogP in the appropriate range also proved helpful in achieving the task. In order to analyze the effectiveness of the proposed algorithm, the molecules were split into buckets based on the binding affinity and LogP and the total number of molecules in each bucket was then compared to the number of molecules identified by MO-MEMES.

**Figure 3:**
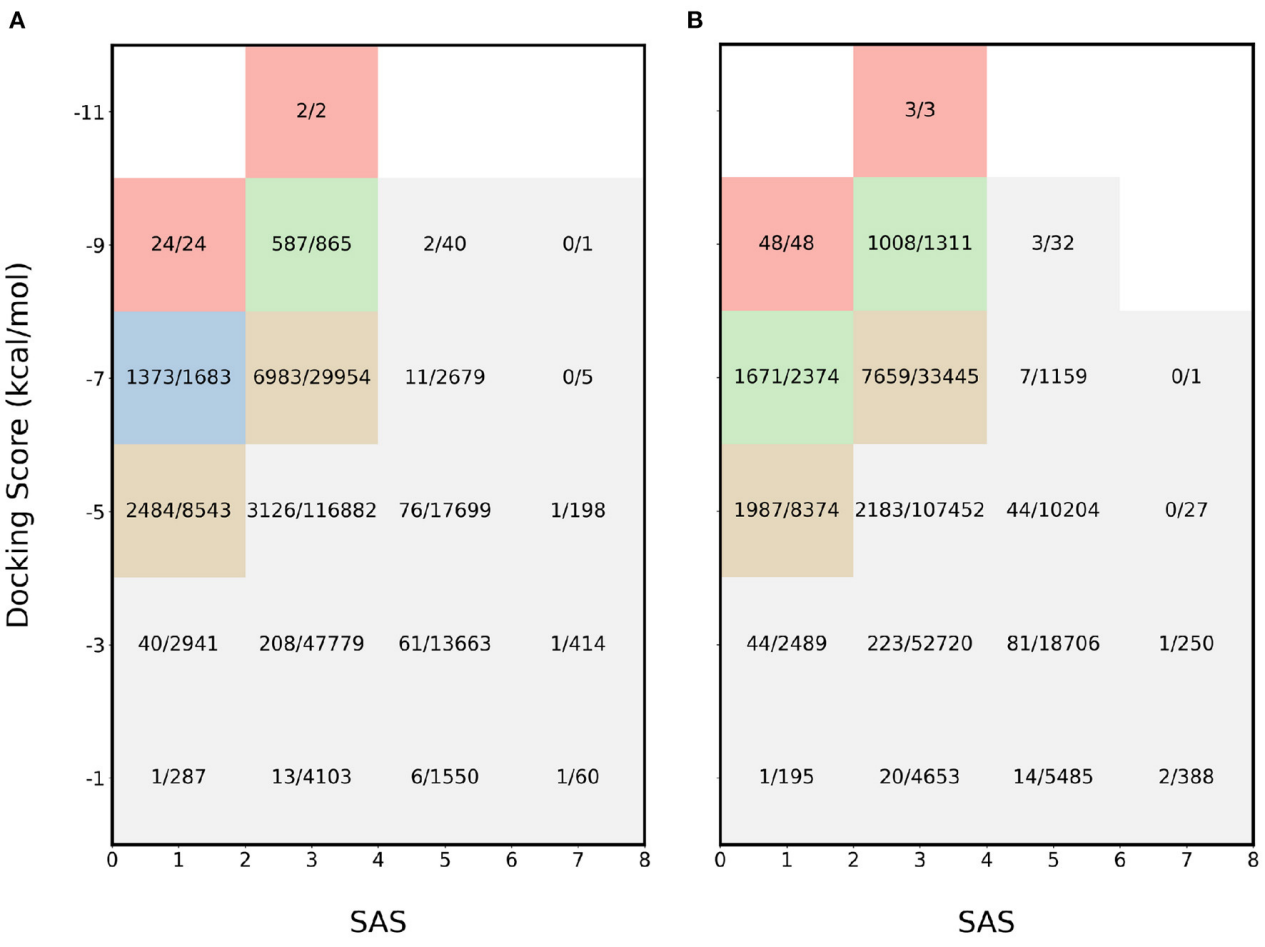
Heatmap of fraction of molecules sampled using MEMES framework from each bucket of docking score and SAS. **(A)** shows the heatmap for protein target 4BTK while **(B)** shows for 6LU7.

For further validation, another combination of properties was used: SAS and binding affinity. The results for the experiments involving binding affinity and SAS for 4BTK and 6LU7 are available in Figures 3A,B, respectively. In this scenario, the most desirable region is the top left and for both proteins, the proposed acquisition function identifies majority of the molecules in the regions where both properties are optimal. In the most desirable region where SAS < 2 and binding affinity < −8 kcal/mol, all the molecules are sampled. This shows that the proposed acquisition function captures the joint information from each property and finds the molecules at the pareto frontier.

#### 3.1.2. Optimizing three properties

The previous section shows the capability of the proposed framework to sample molecules with two properties but for further generalization, the pipeline was used for three properties as well: binding affinity, LogP and SAS. Moreover, from the experiments listed in Table 1, a consistent trend was seen that models using CDDD embeddings performed better than the ones with Mol2vec embeddings. Hence, for this task CDDD embeddings have been used for finding potential hits against both 6LU7 and 4BTK.

**Figure 4:**
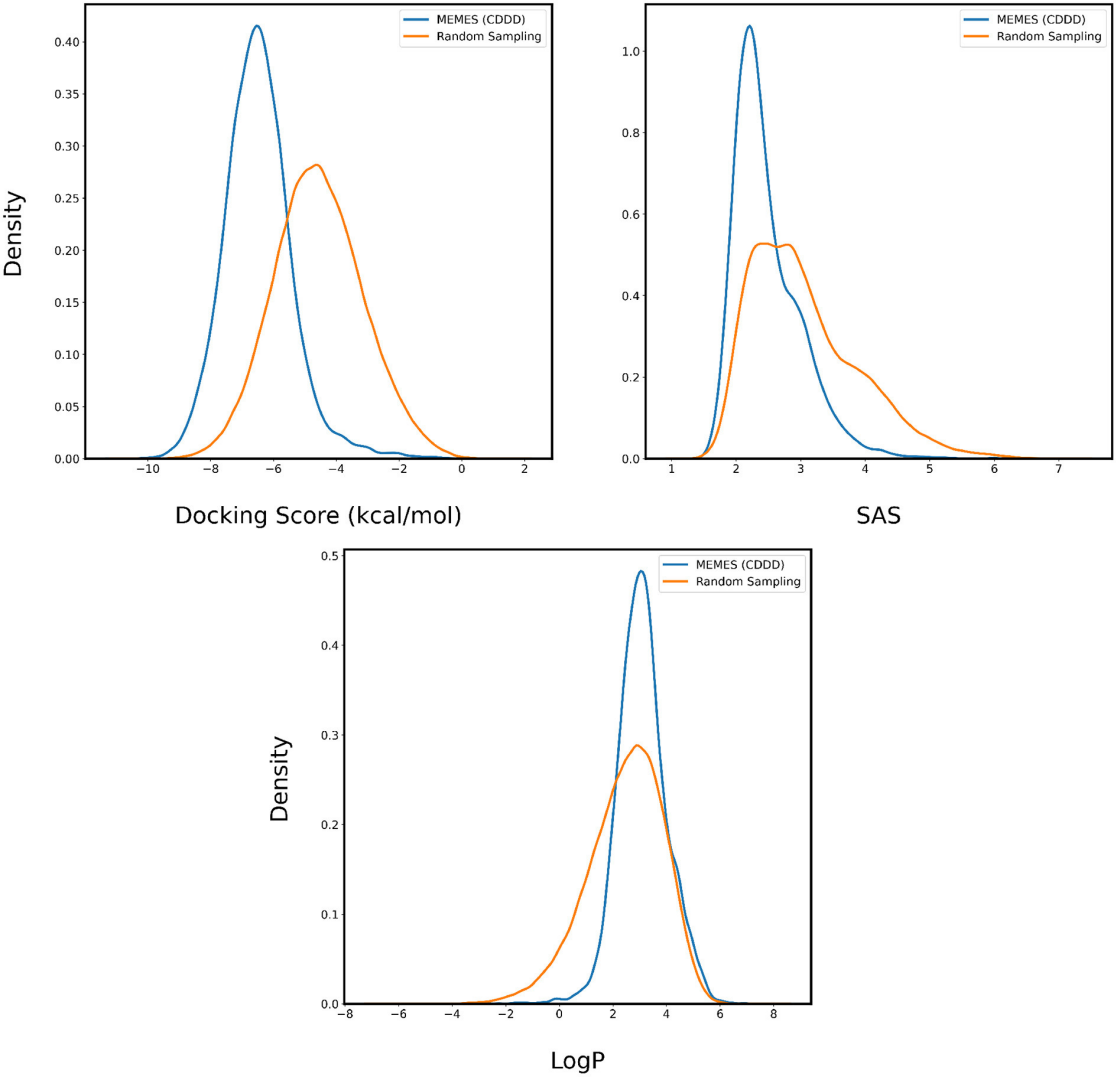
Distribution of binding affinity with 4BTK, SAS, and LogP of molecules sampled by Exact MO-MEMES (blue) and molecules sampled randomly (orange).

**Figure 5:**
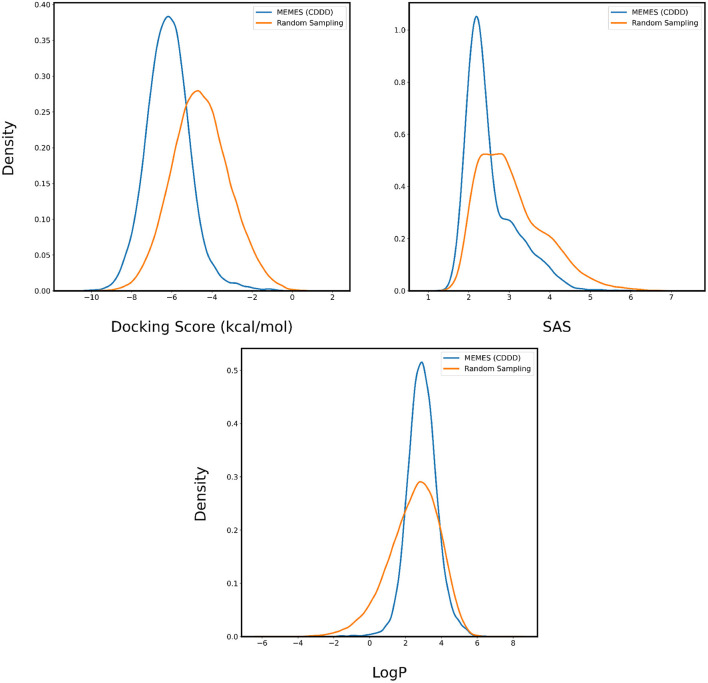
Distribution of binding affinity with 6LU7, SAS, and LogP of molecules sampled by Exact MO-MEMES (blue) and molecules sampled randomly (orange).

In Figures 4, 5, the distributions of binding affinity, LogP and SAS of the sampled molecules are plotted in blue and this is compared to random sampling drawn in orange. The shift in distribution of binding affinity and SAS toward lower values along with a peak close to 2.5 for LogP in comparison to random sampling show that MO-MEMES achieves the goal of sampling molecules with more than two desirable properties as well.

### 3.2. Deep MO-MEMES

The Exact MO-MEMES framework performs extremely well across all the tasks however, Exact MO-MEMES cannot be scaled to very large datasets due to the space and time constraint of the ExactGP model. Hence, the performance of Deep MO-MEMES is first verified on three properties on the ZINC-250K dataset followed by applying it on the enamine collection by HTS which contains 2 million molecules. The application of Deep MO-MEMES to all the experiments listed in Table 1 are available in the Supplementary material.

#### 3.2.1. Optimizing three properties

**Figure 6:**
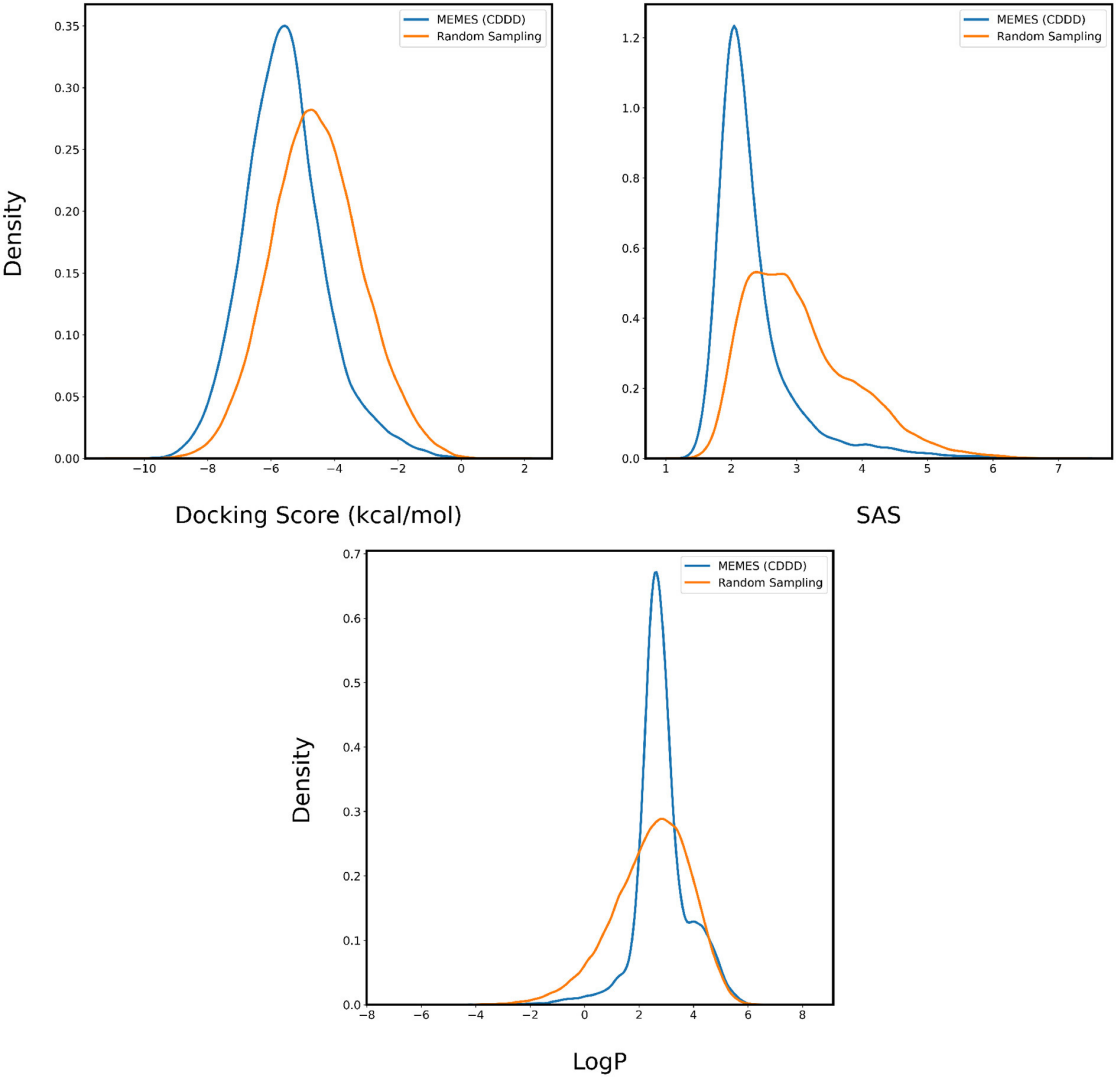
Distribution of binding affinity with 4BTK, SAS, and LogP of molecules sampled by Deep MO-MEMES (blue) and molecules sampled randomly (orange) from the ZINC-250K dataset.

**Figure 7:**
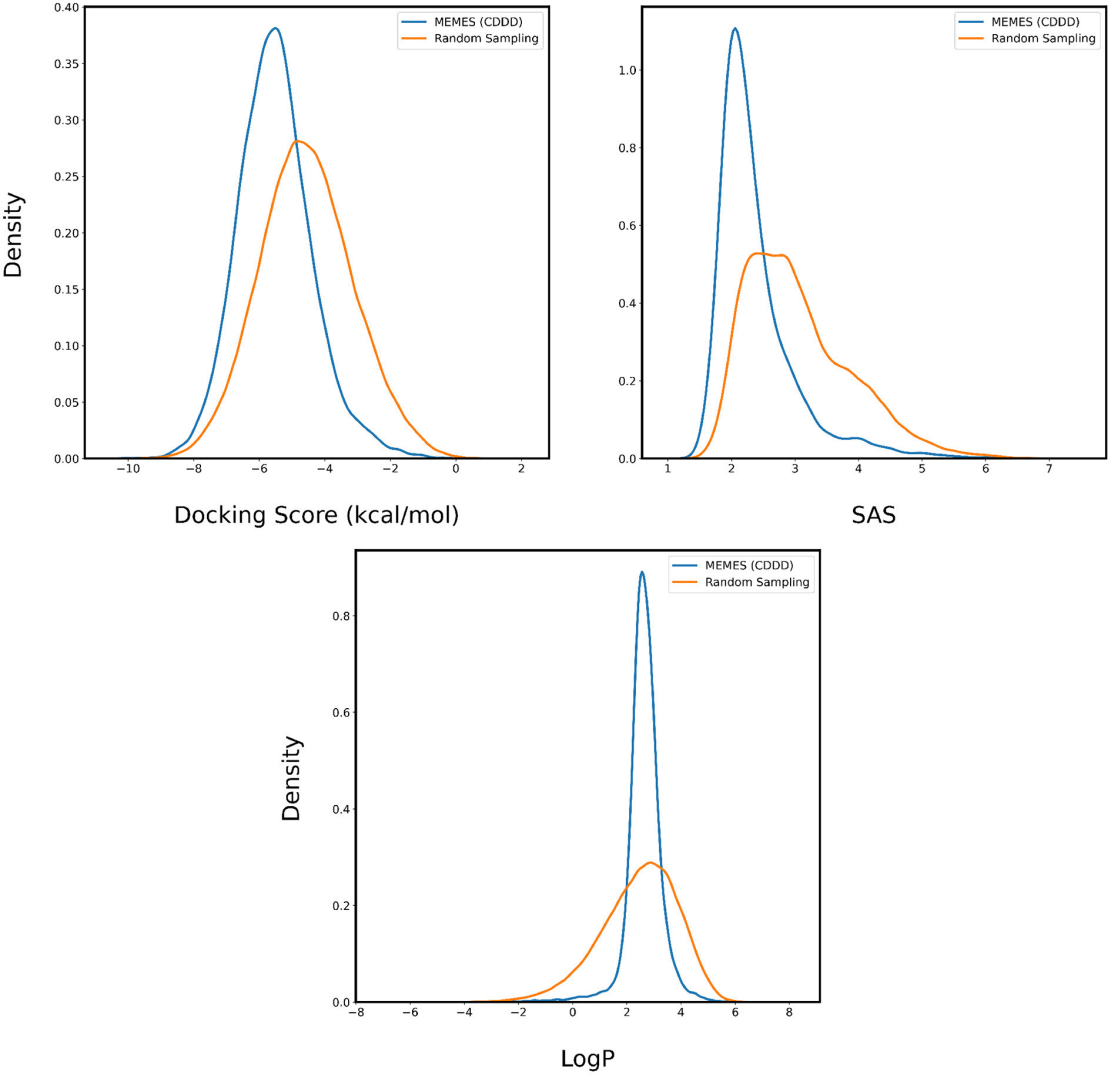
Distribution of binding affinity with 6LU7, SAS, and LogP of molecules sampled by Deep MO-MEMES (blue) and molecules sampled randomly (orange).

The performance is evaluated by applying it to sampling molecules from ZINC-250K that possess a desirable binding affinity, LogP and SAS. In Figures 6, 7, for each property two are plotted. The orange curve represents the distribution of the property of molecules sampled randomly and the blue curve represents the distribution of the property of molecules sampled by Deep MO-MEMES. It is visible that the distribution of binding affinity of molecules sampled by Deep MO-MEMES is further to the left in comparison to random sampling. Similarly, for SAS and LogP, sharp peaks are seen at 2 and 2.5 respectively which is desirable.

#### 3.2.2. Performance on large dataset

In this section, application of Deep MO-MEMES is shown on Enamine^1^ dataset, used for virtual screening. Enamine HTS collection containing 2,106,952 screening compounds is used to find potential hit molecules against target receptor TTBK1.

**Figure 8:**
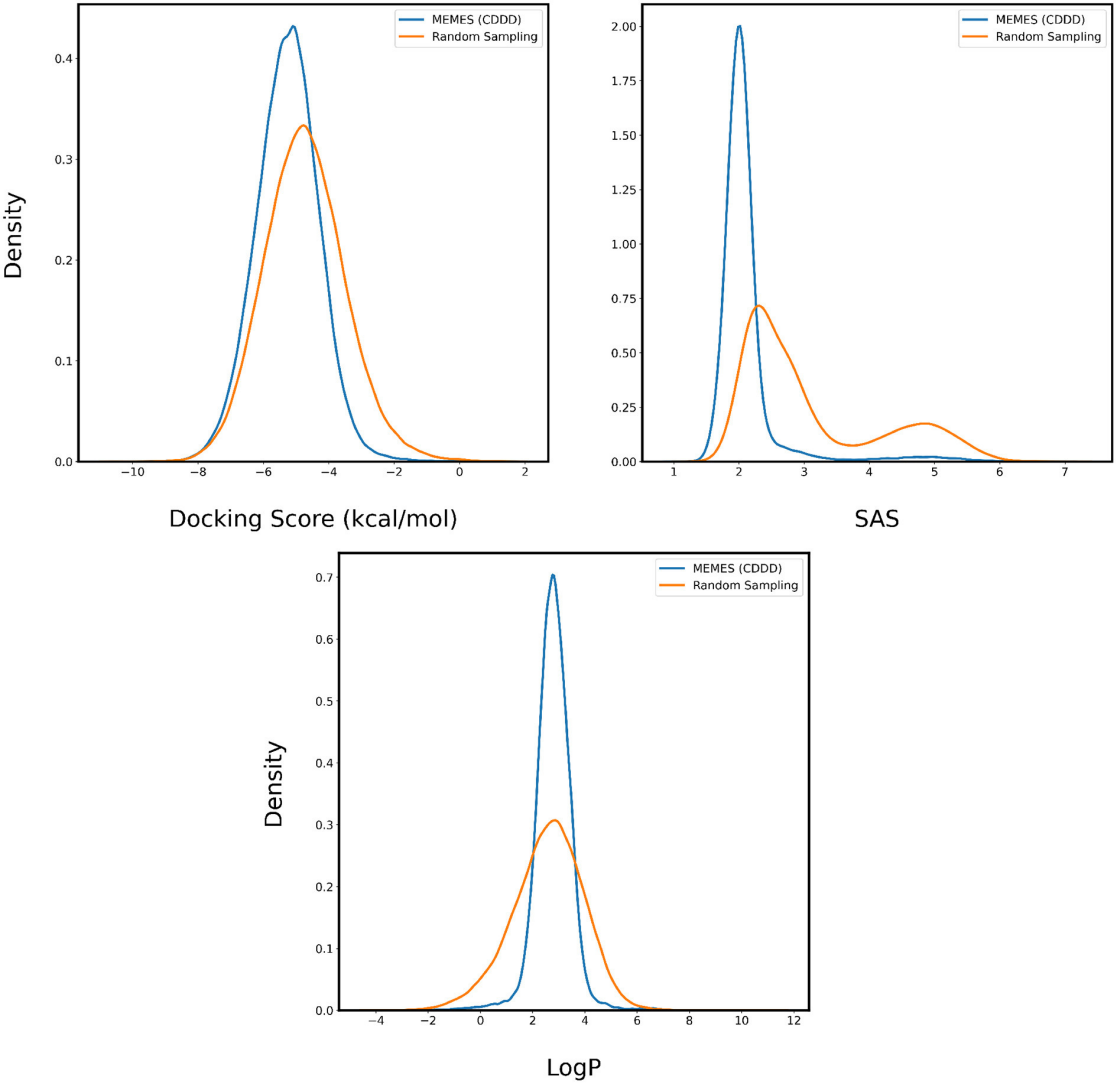
Distribution of binding affinity with 4BTK, SAS, and LogP of molecules sampled by Deep MO-MEMES (blue) and molecules sampled randomly (orange) from the HTS collection by Enamine.

The performance of Deep MO-MEMES is showcased in Figure 8 where we see a trend consistent with the previous sections. The molecules sampled by MO-MEMES possess a more negative docking score, significantly lower SAS and LogP values between 0 and 5. Hence, this shows that MO-MEMES gives great performance on large datasets with multiple properties as well and hence, can be used for screening large libraries as well.

## 4. Conclusion

In this study, we propose MO-MEMES, a multi objective extension of the MEMES framework proposed by Mehta et al. for machine learning aided enhanced molecular sampling. MO-MEMES uses multi-objective Bayesian optimization to sample molecules from drug libraries that possess multiple desirable properties like binding affinity, LogP and synthetic accessibility. This is done by training individual gaussian process models for each property and using the product of the individual expected improvements of each property to sample the next set of points. This acquisition function was used with both Exact MO-MEMES and Deep MO-MEMES variations of MEMES on different combinations of properties and proteins. The proposed approach showed great performance in sampling molecules with desirable properties while optimizing for two and three objectives and consistently sampled more than 90% of the top hits i.e., molecules at the pareto frontier with respect to all properties from the drug library of interest. This method can be efficiently used to screen large molecular libraries that are typically not feasible using traditional techniques and can be used in other domains as well by changing the scoring function that the GPR is expected to learn.

## Data availability statement

The code for MO-MEMES is available at https://github.com/devalab/MO-MEMES. Data can be obtained from corresponding author upon request.

## Author contributions

UDP conceptualized the problem and supervised the project. SM, MG, and UDP designed the ML methodology and wrote the manuscript. SM and MG performed the investigations and data analysis. All authors reviewed the manuscript. All authors contributed to the article and approved the submitted version.

## Conflict of interest

The use of original MEMES framework based on which MO-MEMES has been developed is filed as a US Non-provisional application with the USPTO for the use of MEMES framework in high-throughput screening exercises by the International Institute of Information Technology, Hyderabad. US Application No.: 17526712. The funders did not have any role in the design, idea, data collection, analysis, interpretation, writing of the manuscript or decision to submit it for publication. The authors declare that the research was conducted in the absence of any commercial or financial relationships that could be construed as a potential conflict of interest.

## Publisher's note

All claims expressed in this article are solely those of the authors and do not necessarily represent those of their affiliated organizations, or those of the publisher, the editors and the reviewers. Any product that may be evaluated in this article, or claim that may be made by its manufacturer, is not guaranteed or endorsed by the publisher.

## Footnotes

^1^ https://enamine.net/compound-collections/screening-collection/hts-collection

## References

[1] Schmidt HR Betz RM Dror RO Kruse AC. Structural basis for σ1 receptor ligand recognition. Nat Struct Mol Biol. (2018) 25:981–7. 10.1038/s41594-018-0137-2 30291362 PMC6261271

[2] Lyne PD. Structure-based virtual screening: an overview. Drug Discov Tdy. (2002) 7:1047–55. 10.1016/S1359-6446(02)02483-2 12546894

[3] Cheng T Li Q Zhou Z Wang Y Bryant SH. Structure-based virtual screening for drug discovery: a problem-centric review. AAPS J. (2012) 14:133–41. 10.1208/s12248-012-9322-0 22281989 PMC3282008

[4] McCorvy JD Butler KV Kelly B Rechsteiner K Karpiak J Betz RM . Structure-inspired design of β-arrestin-biased ligands for aminergic GPCRs. Nat Chem Biol. (2018) 14:126–34. 10.1038/nchembio.2527 29227473 PMC5771956

[5] Irwin JJ Shoichet BK. ZINC- a free database of commercially available compounds for virtual screening. J Chem Inform Model. (2005) 45:177–82. 10.1021/ci049714+ 15667143 PMC1360656

[6] Gaulton A Bellis LJ Bento AP Chambers J Davies M Hersey A . ChEMBL: a large-scale bioactivity database for drug discovery. Nucleic Acids Res. (2012) 40:D1100–7. 10.1093/nar/gkr777 21948594 PMC3245175

[7] Pathak Y Laghuvarapu S Mehta S Priyakumar UD. Chemically interpretable graph interaction network for prediction of pharmacokinetic properties of drug-like molecules. In: Proceedings of the AAAI Conference on Artificial Intelligence. (2020). p. 873–80. 10.1609/aaai.v34i01.5433

[8] Laghuvarapu S Pathak Y Priyakumar UD. Band nn: A deep learning framework for energy prediction and geometry optimization of organic small molecules. J Comput Chem. (2020) 41:790–9. 10.1002/jcc.26128 31845368

[9] Wu Z Ramsundar B Feinberg EN Gomes J Geniesse C Pappu AS . MoleculeNet: a benchmark for molecular machine learning. Chem Sci. (2018) 9:513–30. 10.1039/C7SC02664A 29629118 PMC5868307

[10] Vamathevan J Clark D Czodrowski P Dunham I Ferran E Lee G . Applications of machine learning in drug discovery and development. Nat Rev Drug Discov. (2019) 18:463–77. 10.1038/s41573-019-0024-5 30976107 PMC6552674

[11] Su H Wang W Du Z Peng Z Gao SH Cheng MM . Improved protein structure prediction using a new multi-scale network and homologous templates. Adv Sci. (2021) 2021:2102592. 10.1002/advs.202102592 34719864 PMC8693034

[12] Jumper J Evans R Pritzel A Green T Figurnov M Ronneberger O . Highly accurate protein structure prediction with AlphaFold. Nature. (2021) 596:583–9. 10.1038/s41586-021-03819-2 34265844 PMC8371605

[13] Noé F Tkatchenko A Müller KR Clementi C. Machine learning for molecular simulation. Annu Rev Phys Chem. (2020) 71:361–90. 10.1146/annurev-physchem-042018-052331 32092281

[14] Pattnaik P Raghunathan S Kalluri T Bhimalapuram P Jawahar C Priyakumar UD. Machine learning for accurate force calculations in molecular dynamics simulations. J Phys Chem A. (2020) 124:6954–67. 10.1021/acs.jpca.0c03926 32786995

[15] Manzhos S Carrington T Jr. Neural network potential energy surfaces for small molecules and reactions. Chem Rev. (2020) 121:10187–217. 10.1021/acs.chemrev.0c00665 33021368

[16] Aggarwal R Gupta A Chelur V Jawahar C Priyakumar UD. Deeppocket: ligand binding site detection and segmentation using 3d convolutional neural networks. J Chem Inform Model. (2021) 10.26434/chemrxiv.14611146 34374539

[17] Bagal V Aggarwal R Vinod P Priyakumar UD. MolGPT: molecular generation using a transformer-decoder model. J Chem Inform Model. (2021) 62:2064–76. 10.26434/chemrxiv.14561901 34694798

[18] Podda M Bacciu D Micheli A. A deep generative model for fragment-based molecule generation. In: International Conference on Artificial Intelligence and Statistics. PMLR (2020). p. 2240–50.

[19] Grisoni F Moret M Lingwood R Schneider G. Bidirectional molecule generation with recurrent neural networks. J Chem Inform Model. (2020) 60:1175–83. 10.1021/acs.jcim.9b00943 31904964

[20] Kusner MJ Paige B Hernández-Lobato JM. Grammar variational autoencoder. In: International Conference on Machine Learning. PMLR (2017). p. 1945–54.

[21] Jin W Barzilay R Jaakkola T. Junction tree variational autoencoder for molecular graph generation. In: International Conference on Machine Learning. PMLR (2018). p. 2323–32.

[22] Lim J Ryu S Kim JW Kim WY. Molecular generative model based on conditional variational autoencoder for *de novo* molecular design. J Cheminform. (2018) 10:1–9. 10.1186/s13321-018-0286-7 29995272 PMC6041224

[23] Guimaraes GL Sanchez-Lengeling B Outeiral C Farias PLC Aspuru-Guzik A. Objective-reinforced generative adversarial networks (ORGAN) for sequence generation models. arXiv preprint arXiv:170510843. (2017). 10.48550/arXiv.1705.10843

[24] De Cao N Kipf T. MolGAN: an implicit generative model for small molecular graphs. arXiv preprint arXiv:180511973. (2018). 10.48550/arXiv.1805.11973

[25] Prykhodko O Johansson SV Kotsias PC Arús-Pous J Bjerrum EJ Engkvist O . A *de novo* molecular generation method using latent vector based generative adversarial network. J Cheminform. (2019) 11:1–13. 10.1186/s13321-019-0397-9 33430938 PMC6892210

[26] Maziarka Ł Pocha A Kaczmarczyk J Rataj K Danel T Warchoł M. Mol-CycleGAN: a generative model for molecular optimization. J Cheminform. (2020) 12:1–18. 10.1186/s13321-019-0404-1 33431006 PMC6950853

[27] Popova M Isayev O Tropsha A. Deep reinforcement learning for *de novo* drug design. Sci Adv. (2018) 4:eaap7885. 10.1126/sciadv.aap7885 30050984 PMC6059760

[28] You J Liu B Ying Z Pande V Leskovec J. Graph convolutional policy network for goal-directed molecular graph generation. In: 32nd Conference on Neural Information Processing Systems. Montreal, QC (2018).

[29] Khemchandani Y O'Hagan S Samanta S Swainston N Roberts TJ Bollegala D . DeepGraphMolGen, a multi-objective, computational strategy for generating molecules with desirable properties: a graph convolution and reinforcement learning approach. J Cheminform. (2020) 12:1–17. 10.1186/s13321-020-00454-3 33431037 PMC7487898

[30] Goel M Raghunathan S Laghuvarapu S Priyakumar UD. MoleGuLAR: molecule generation using reinforcement learning with alternating rewards. J Chem Inform Model. (2021) 61:5815–26. 10.1021/acs.jcim.1c01341 34866384

[31] Gao W Coley CW. The synthesizability of molecules proposed by generative models. J Chem Inform Model. (2020) 60:5714–23. 10.1021/acs.jcim.0c00174 32250616

[32] Tomberg A Boström J. Can “easy” chemistry produce complex, diverse and novel molecules? Drug Discover Today. (2020) 25:2174–81. 10.26434/chemrxiv.12563231 33010477

[33] Lyu J Wang S Balius TE Singh I Levit A Moroz YS . Ultra-large library docking for discovering new chemotypes. Nature. (2019) 566:224–9. 10.1038/s41586-019-0917-9 30728502 PMC6383769

[34] Irwin JJ Tang KG Young J Dandarchuluun C Wong BR Khurelbaatar M . ZINC20–a free ultralarge-scale chemical database for ligand discovery. J Chem Inform Model. (2020) 60:6065–73. 10.1021/acs.jcim.0c00675 33118813 PMC8284596

[35] Liao Z You R Huang X Yao X Huang T Zhu S. DeepDock: enhancing ligand-protein interaction prediction by a combination of ligand and structure information. In: 2019 IEEE International Conference on Bioinformatics and Biomedicine (BIBM). San Diego, CA: IEEE (2019). p. 311–7. 10.1109/BIBM47256.2019.8983365

[36] Graff DE Shakhnovich EI Coley CW. Accelerating high-throughput virtual screening through molecular pool-based active learning. Chem Sci. (2021) 12:7866–81. 10.1039/D0SC06805E 34168840 PMC8188596

[37] Gupta A Zhou HX. Machine learning-enabled pipeline for large-scale virtual drug screening. J Chem Inform Model. (2021) 61:4236–44. 10.1021/acs.jcim.1c00710 34399578 PMC8478848

[38] Baird SG Diep TQ Sparks TD. DiSCoVeR: a materials discovery screening tool for high performance, unique chemical compositions. Digit Discov. (2022) 10.33774/chemrxiv-2021-5l2f8-v3

[39] Mehta S Laghuvarapu S Pathak Y Sethi A Alvala M Priyakumar UD. Memes: machine learning framework for enhanced molecular screening. Chem Sci. (2021) 12:11710–21. 10.1039/D1SC02783B 34659706 PMC8442698

[40] Daulton S Balandat M Bakshy E. Differentiable expected hypervolume improvement for parallel multi-objective Bayesian optimization. In: The Conference on Uncertainty in Artificial Intelligence (UAI). Elndhoven (2020). p. 9851–64.

[41] Daulton S Eriksson D Balandat M Bakshy E. Multi-objective bayesian optimization over high-dimensional search spaces. arXiv preprint arXiv:210910964. (2021).

[42] Morris GM Huey R Lindstrom W Sanner MF Belew RK Goodsell DS . AutoDock4 and AutoDockTools4: automated docking with selective receptor flexibility. J Comput Chem. (2009) 30:2785–91. 10.1002/jcc.21256 19399780 PMC2760638

[43] Sterling T Irwin JJ. ZINC 15-ligand discovery for everyone. J Chem Inform Model. (2015) 55:2324–37. 10.1021/acs.jcim.5b00559 26479676 PMC4658288

[44] Jaeger S Fulle S Turk S. Mol2vec: unsupervised machine learning approach with chemical intuition. J Chem Inform Model. (2018) 58:27–35. 10.1021/acs.jcim.7b00616 29268609

[45] Winter R Montanari F Noé F Clevert DA. Learning continuous and data-driven molecular descriptors by translating equivalent chemical representations. Chem Sci. (2019) 10:1692–701. 10.1039/C8SC04175J 30842833 PMC6368215

[46] Gardner J Pleiss G Weinberger KQ Bindel D Wilson AG. Gpytorch: blackbox matrix-matrix gaussian process inference with GPU acceleration. In: Advances in Neural Information Processing Systems. (2018).

[47] Brochu E Cora VM De Freitas N. A tutorial on Bayesian optimization of expensive cost functions, with application to active user modeling and hierarchical reinforcement learning. arXiv preprint arXiv:10122599. (2010). Available online at: https://proceedings.mlr.press/v119/suzuki20a.html

[48] Suzuki S Takeno S Tamura T Shitara K Karasuyama M. Multi-objective Bayesian optimization using Pareto-frontier entropy. In: International Conference on Machine Learning. PMLR (2020). p. 9279–88.

[49] Zhang L Lin D Sun X Curth U Drosten C Sauerhering L . Crystal structure of SARS-CoV-2 main protease provides a basis for design of improved α-ketoamide inhibitors. Science. (2020) 368:409–12. 10.1126/science.abb3405 32198291 PMC7164518

[50] Sato S Cerny RL Buescher JL Ikezu T. Tau-tubulin kinase 1 (TTBK1), a neuron-specific tau kinase candidate, is involved in tau phosphorylation and aggregation. J Neurochem. (2006) 98:1573–84. 10.1111/j.1471-4159.2006.04059.x 16923168

