# Supplementary material for: MO-MEMES: A method for accelerating virtual screening using multi-objective Bayesian optimization
Source: Front Med (Lausanne). 2022 Sep 23;9:916481. doi: 10.3389/fmed.2022.916481 (PMC9537730; doi:10.3389/fmed.2022.916481)
Supplement: Supplementary file 1 [file Data_Sheet_1.PDF]

Supplementary Material: Accelerating Virtual Screening using Multi-Objective Bayesian Optimization

1 DEEP GAUSSIAN PROCESSES

ExactGPs perform extremely well in approximating black-box functions but the time complexity of the model is $O(n^3)$ which makes it infeasible for usage on datasets with more than a few hundred thousand datapoints. In such a scenario, deep gaussian processes have proven to be a great alternative (Damianou and Lawrence (2013)).

DeepGPs are a type of deep belief network in which each hidden unit is a gaussian process. The input from each layer is used as the input for the next layer and hence it can be defined as a composition of functions and hence we can define a DeepGP on k points as –

$$f^{(1:L)}(x_{1:k}) = f^{(L)}(f^{(L-1)}(..f^{(2)}(f^{(1)}(x_{1:k}))..)) \tag{S1}$$

where $f_d^{(l)} \sim GP(0, \kappa_d^{(l)}(x, x‘))$ for $f_d^{(l)} \in f^{(l)}$.

In Equation S1, L denotes the total number of layers and each layer has it’s own kernel and the noise between layers is assumed to be an identically and independently distributed gaussian which is a part of the kernel: $\kappa_{noisy}(x_i, x_j) = \kappa(x_i, x_j) + \sigma_l^2 \delta_{ij}$. Here, σ_l^2 is the noise between layers and δ_{ij} is the kronecker delta and the joint probability of the DeepGP is given by:

$$p(y, \{f^{(l)}\}_{l=1}^{(L)}) = \prod_{i=1}^{N} p(y_i | f_i^{(L)}) \prod_{i=1}^{L} p(f_l | f_{l-1}) \tag{S2}$$

The first part of Equation S2, the first term corresponds to the likelihood and the second term denotes the gaussian prior. Non linear transformation is applied on the output of every hidden layer due to which the exact inference is not tractable. To overcome this problem various numbers of approximations have been developed such as expected propagation, variational auto-encoded deep Gaussian processes, and doubly stochastic variational inference for deep Gaussian processes (Bui et al. (2016); Dai et al. (2015); Salimbeni and Deisenroth (2017)). This study uses doubly stochastic variational inference.

2 RESULTS

2.1 Exact MO-MEMES with Mol2vec Embeddings

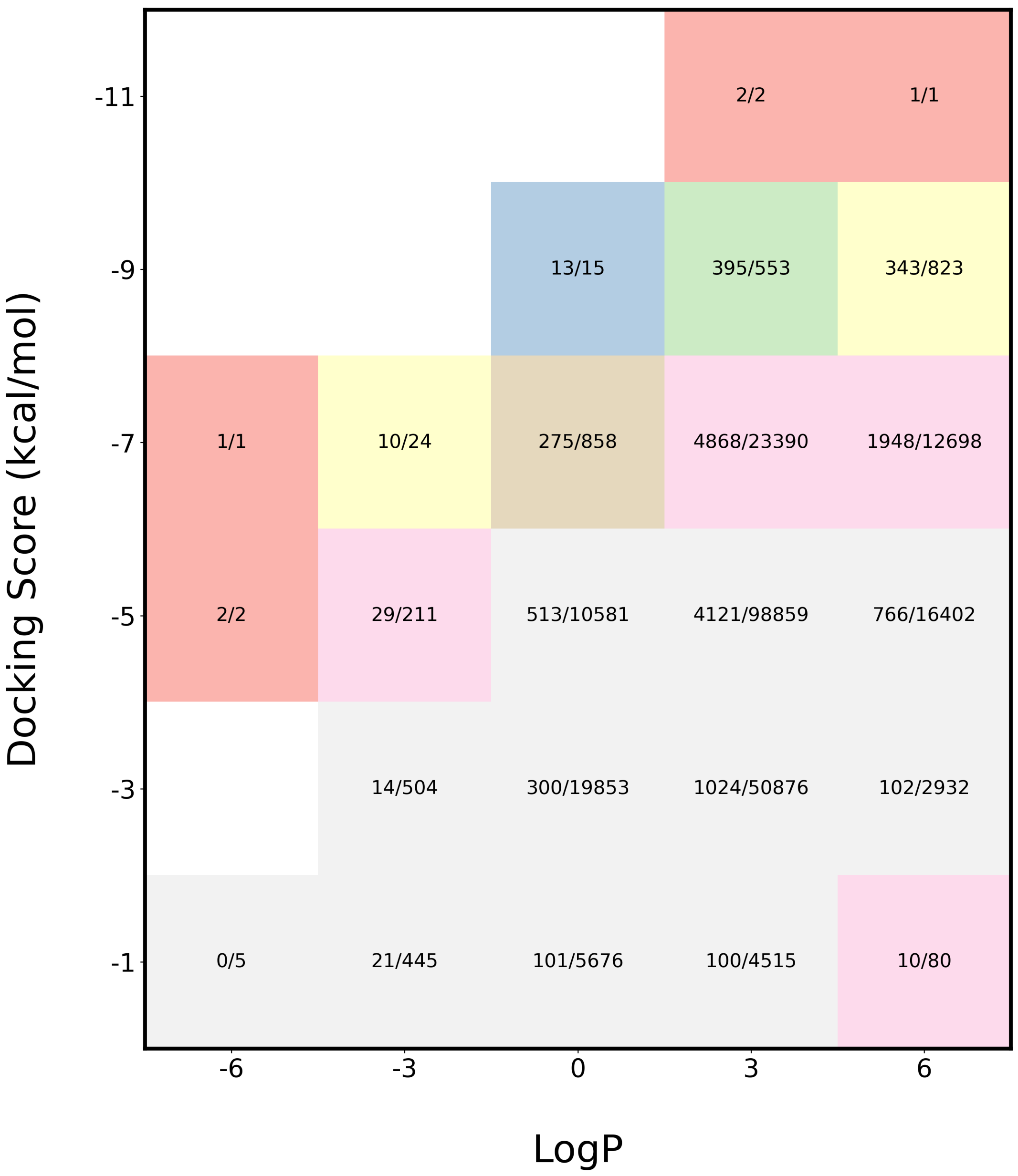

Figure S1. Heatmap of molecules sampled by Exact MO-MEMES using Mol2vec embeddings out of total molecules in each bucket with LogP between 0 and 5 and high binding affinity to 4BTK

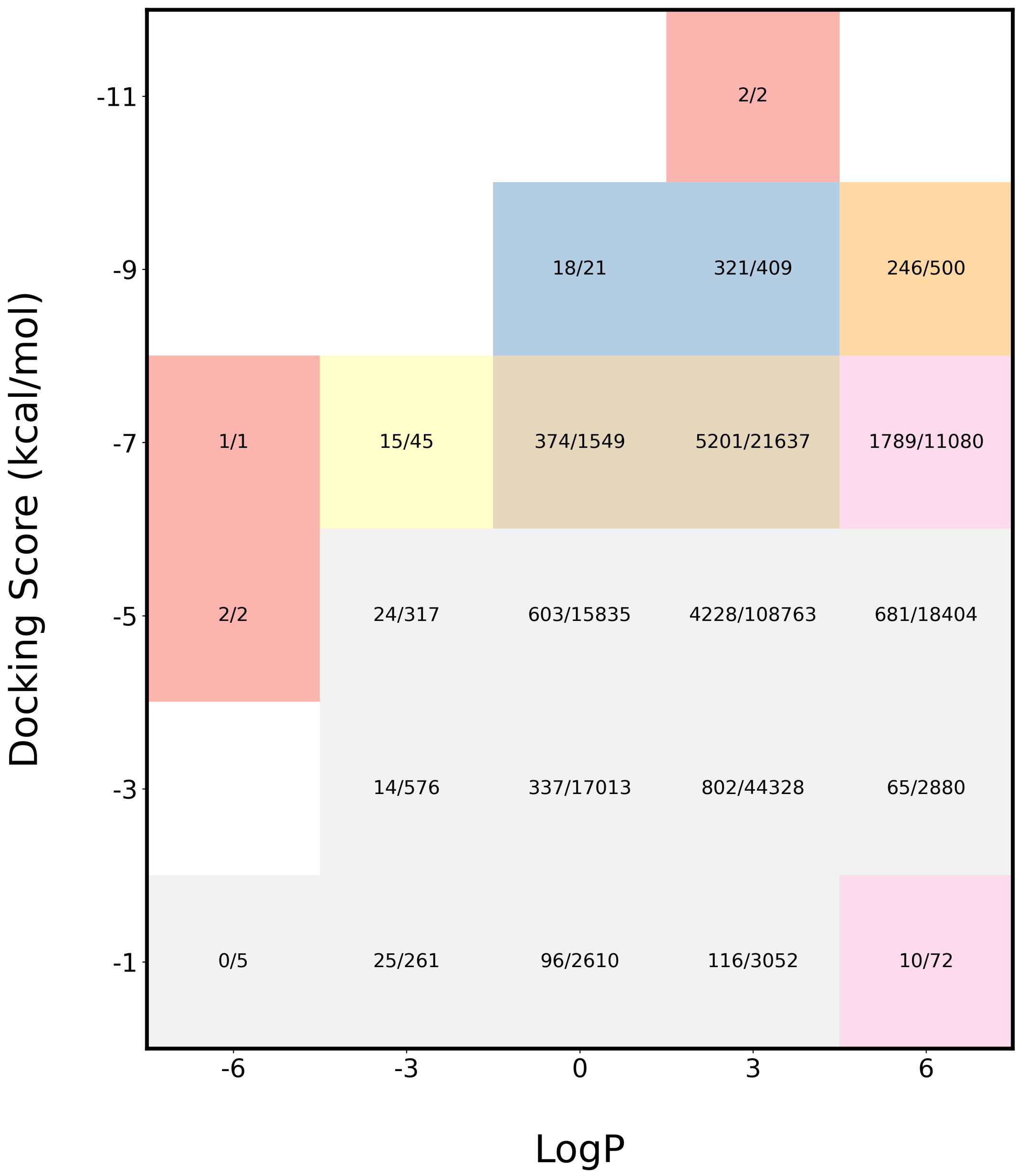

Figure S2. Heatmap of molecules sampled by Exact MO-MEMES using Mol2vec embeddings out of total molecules in each bucket with LogP between 0 and 5 and high binding affinity to 6LU7

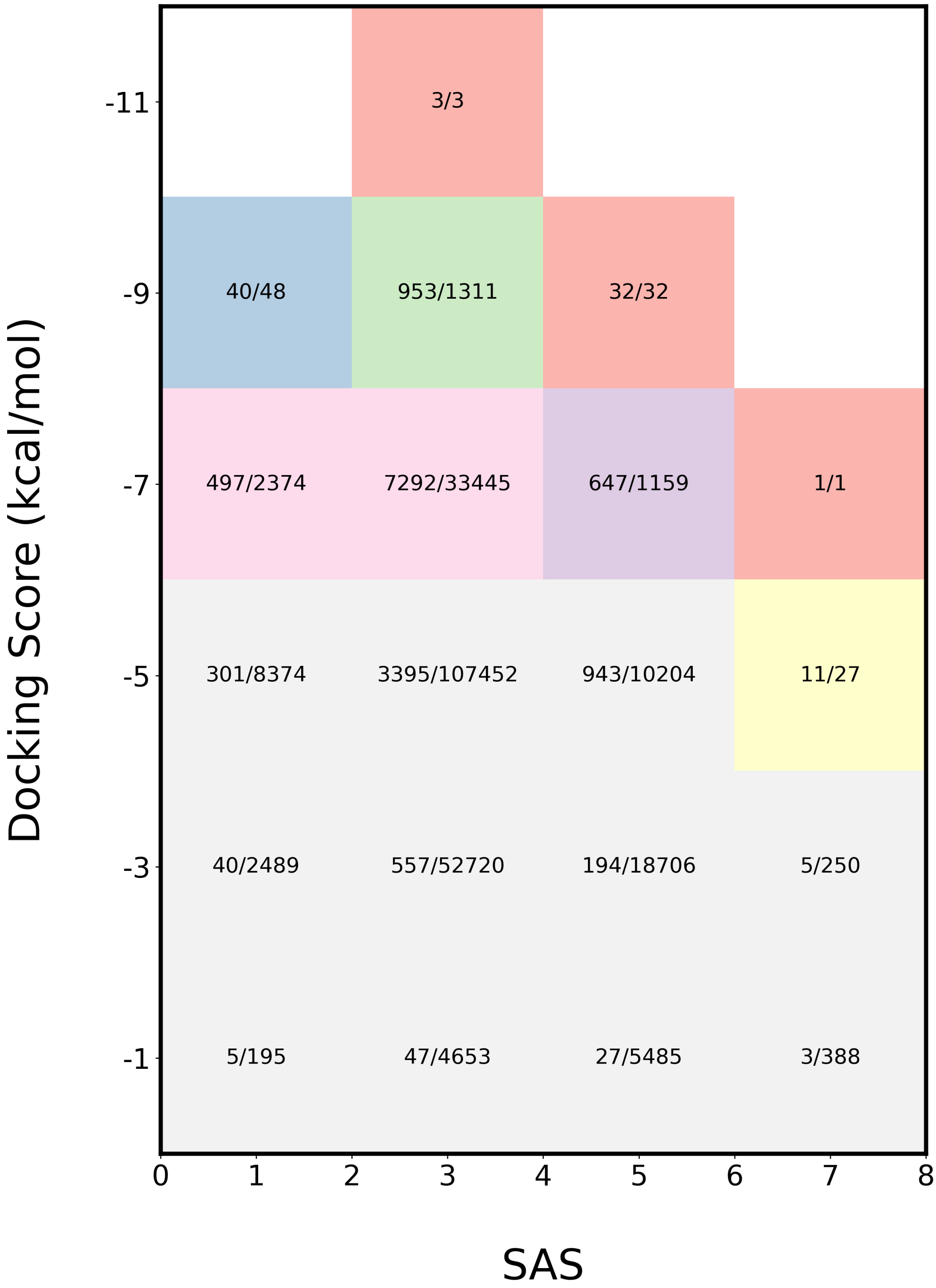

Figure S3. Heatmap of molecules sampled by Exact MO-MEMES using Mol2vec embeddings out of total molecules in each bucket low SAS and high binding affinity to 4BTK

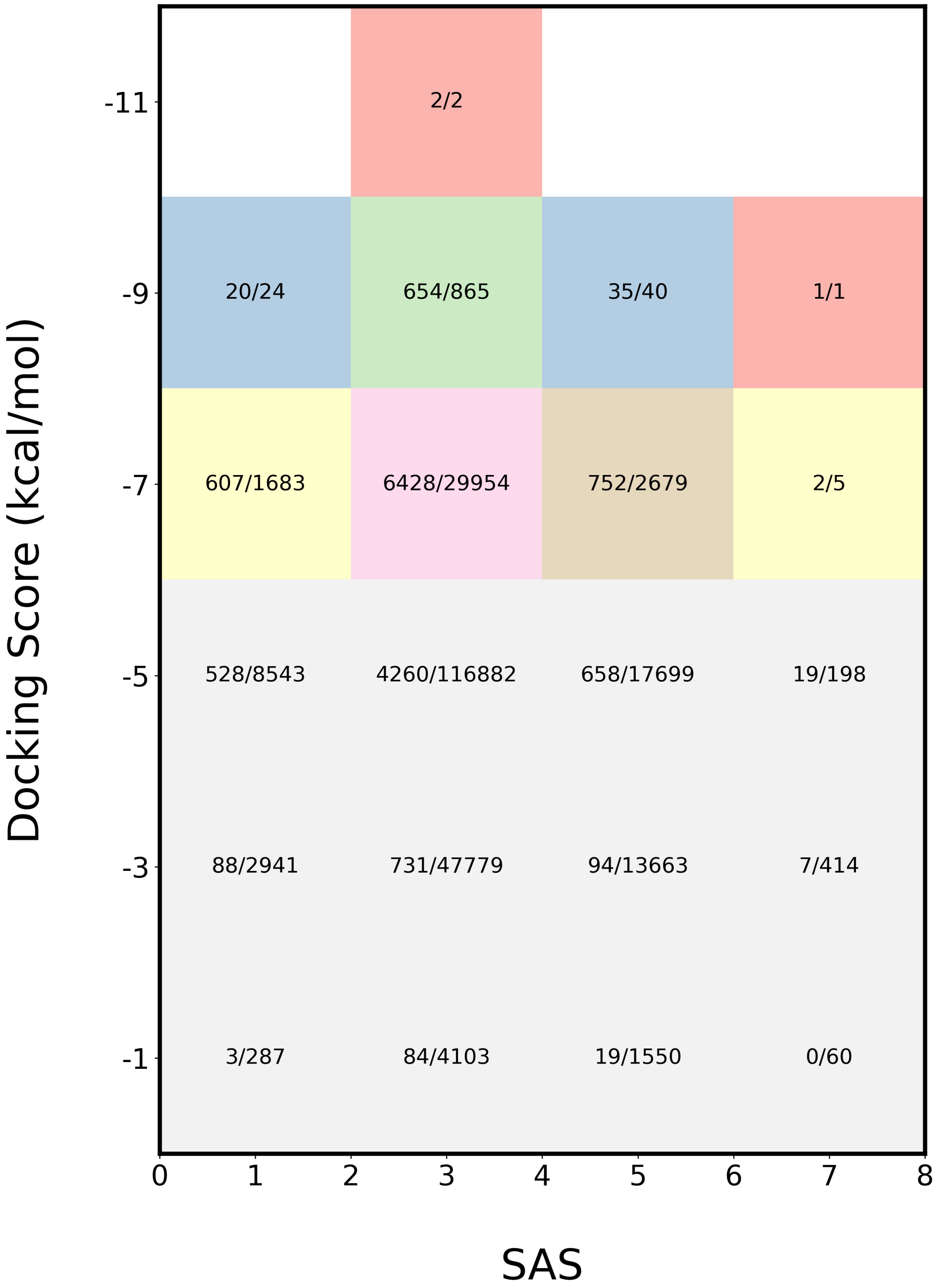

Figure S4. Heatmap of molecules sampled by Exact MO-MEMES using Mol2vec embeddings out of total molecules in each bucket low SAS and high binding affinity to 6LU7

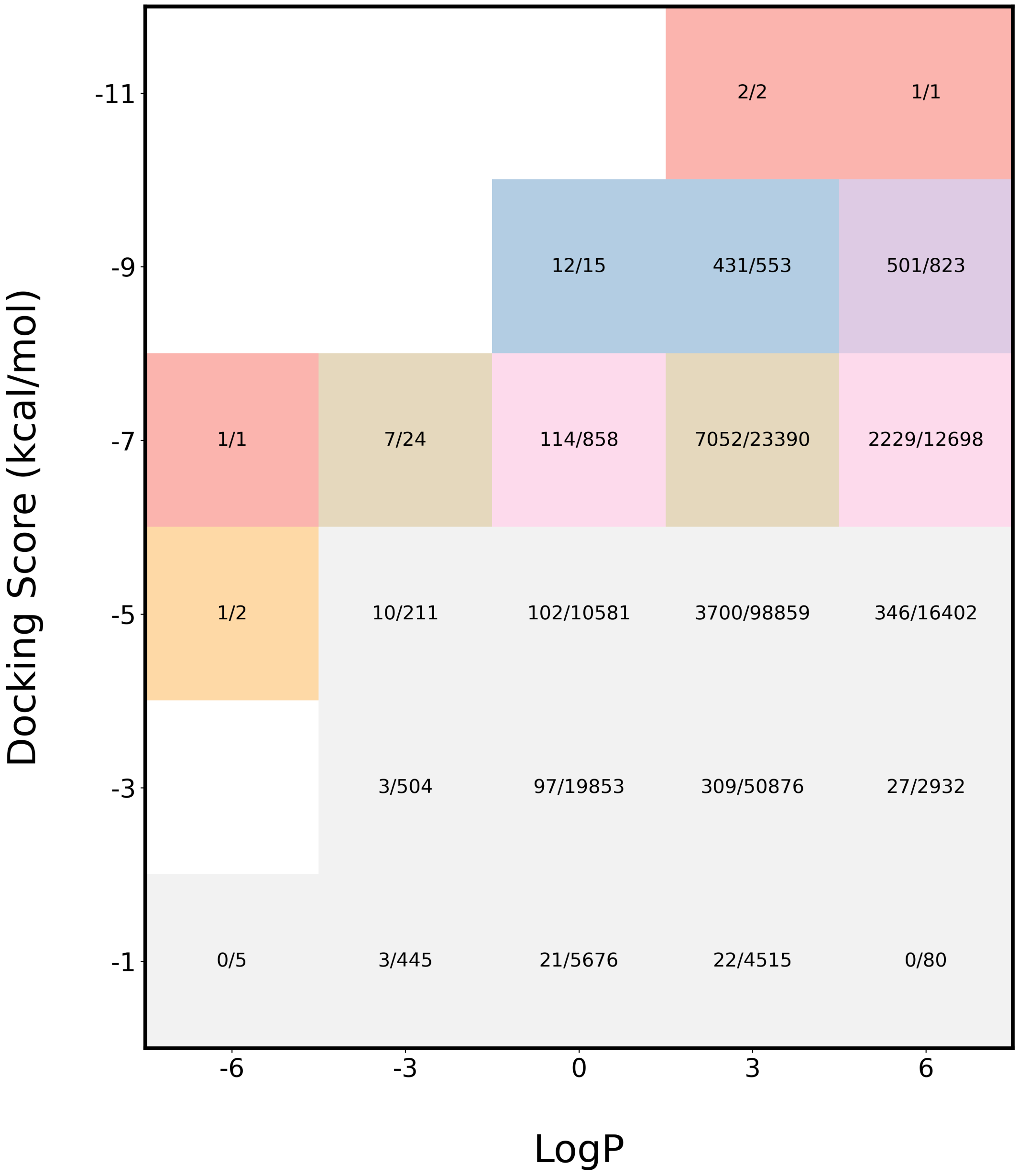

Figure S5. Heatmap of molecules sampled by Deep MO-MEMES using Mol2vec embeddings out of total molecules in each bucket with LogP between 0 and 5 and high binding affinity to 4BTK

2.2 Deep MO-MEMES with Mol2vec Embeddings

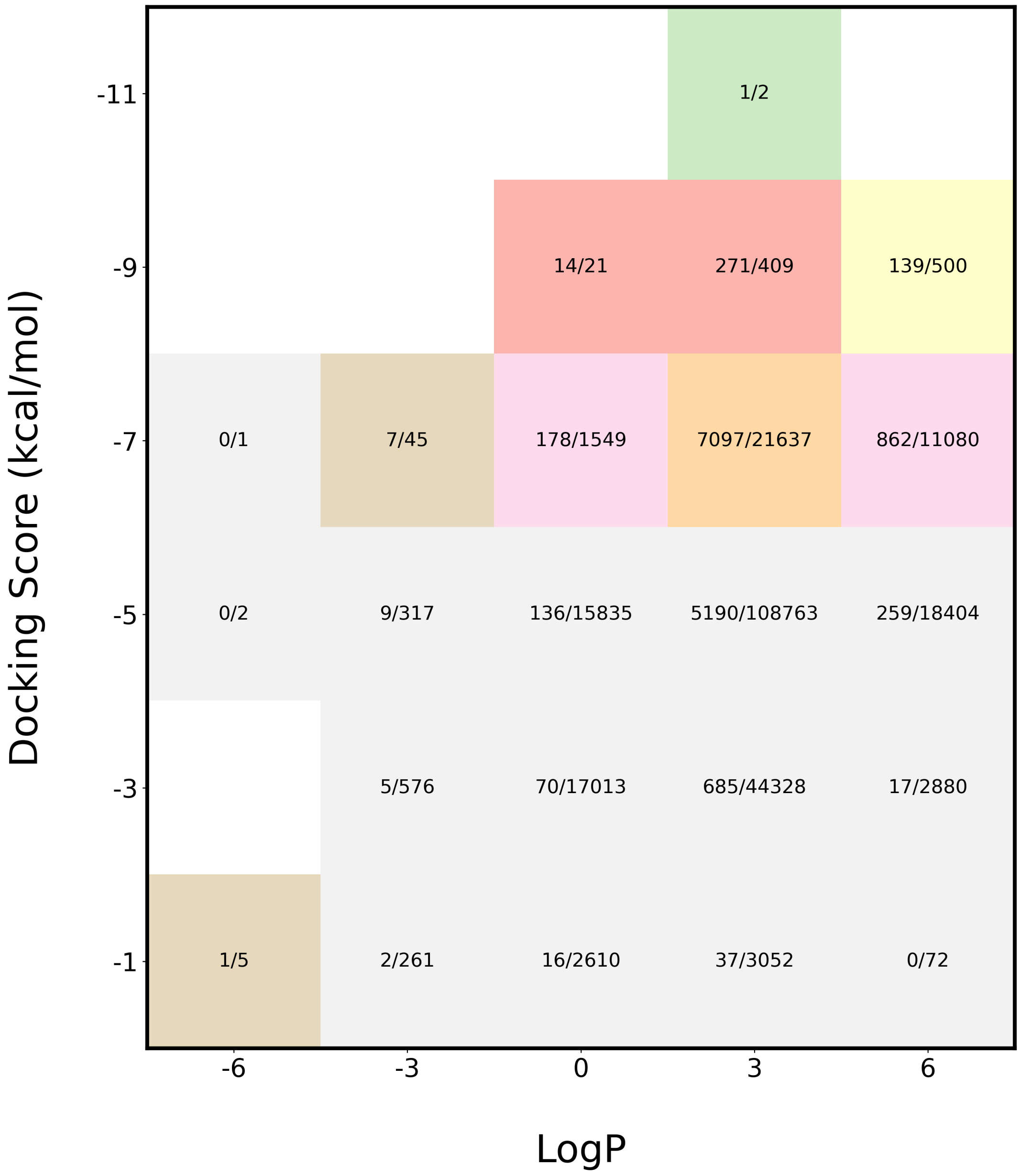

Figure S6. Heatmap of molecules sampled by Deep MO-MEMES using Mol2vec embeddings out of total molecules in each bucket with LogP between 0 and 5 and high binding affinity to 6LU7

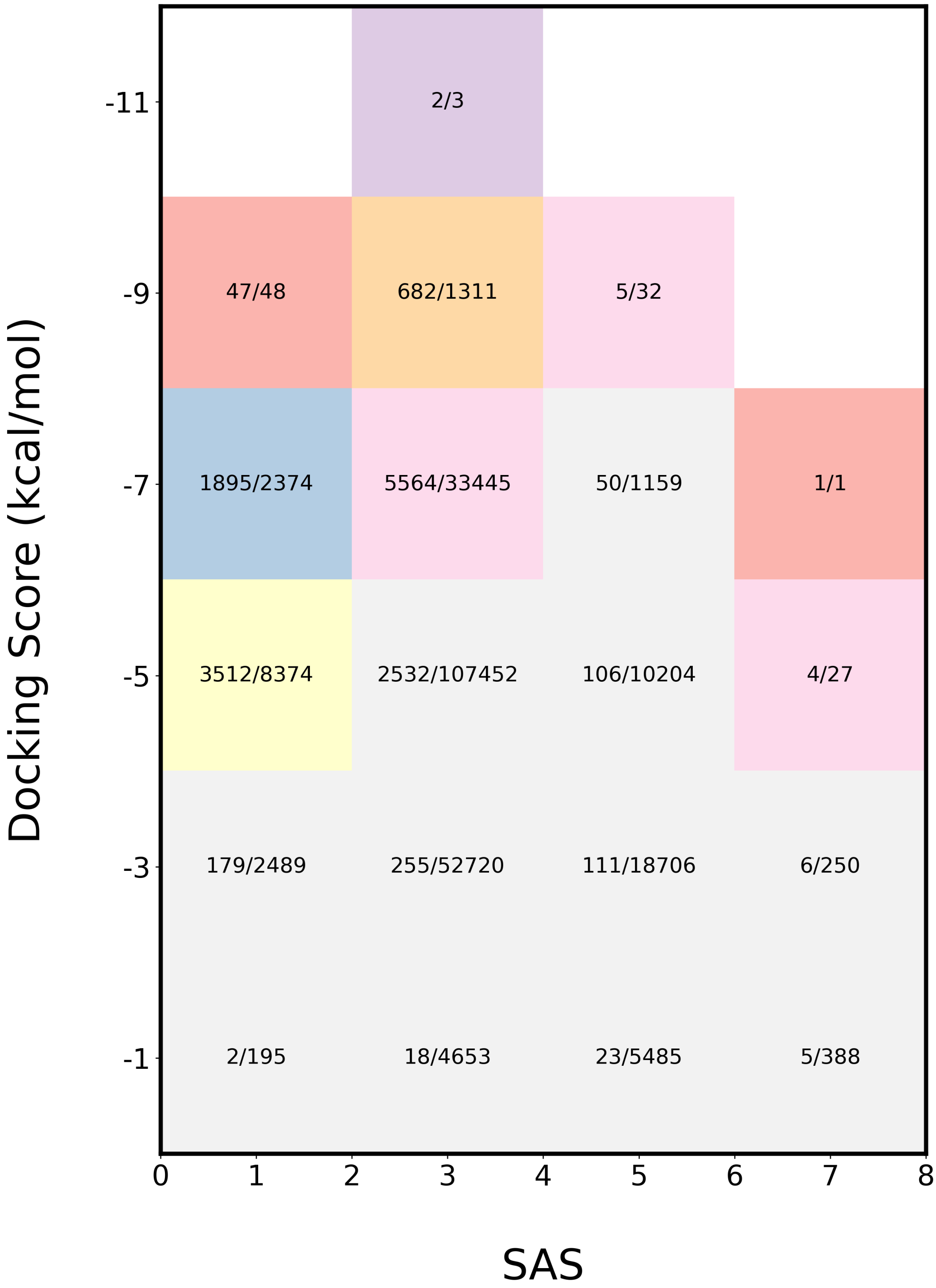

Figure S7. Heatmap of molecules sampled by Deep MO-MEMES using Mol2vec embeddings out of total molecules in each bucket low SAS and high binding affinity to 4BTK

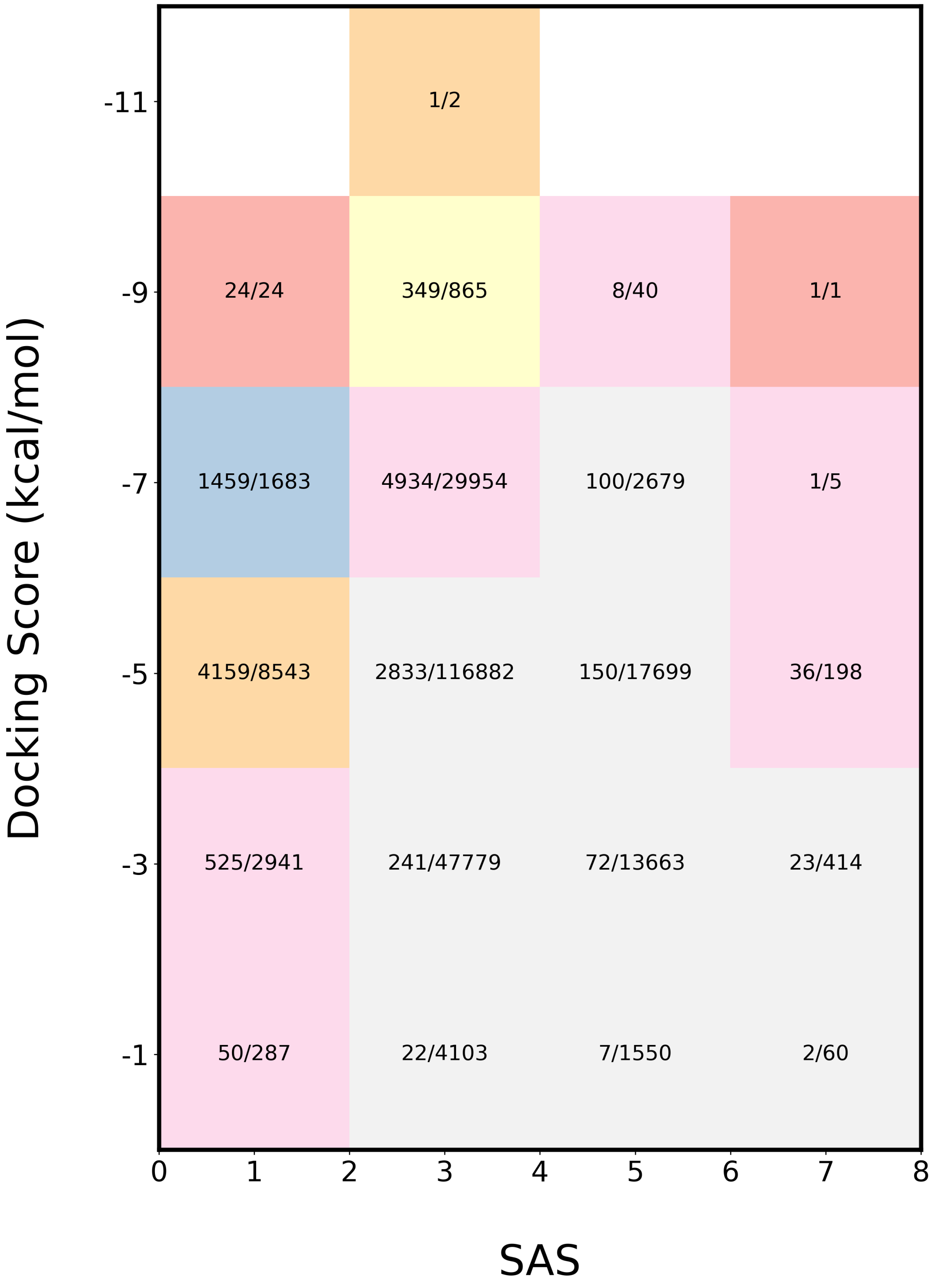

Figure S8. Heatmap of molecules sampled by Deep MO-MEMES using Mol2vec embeddings out of total molecules in each bucket low SAS and high binding affinity to 6LU7

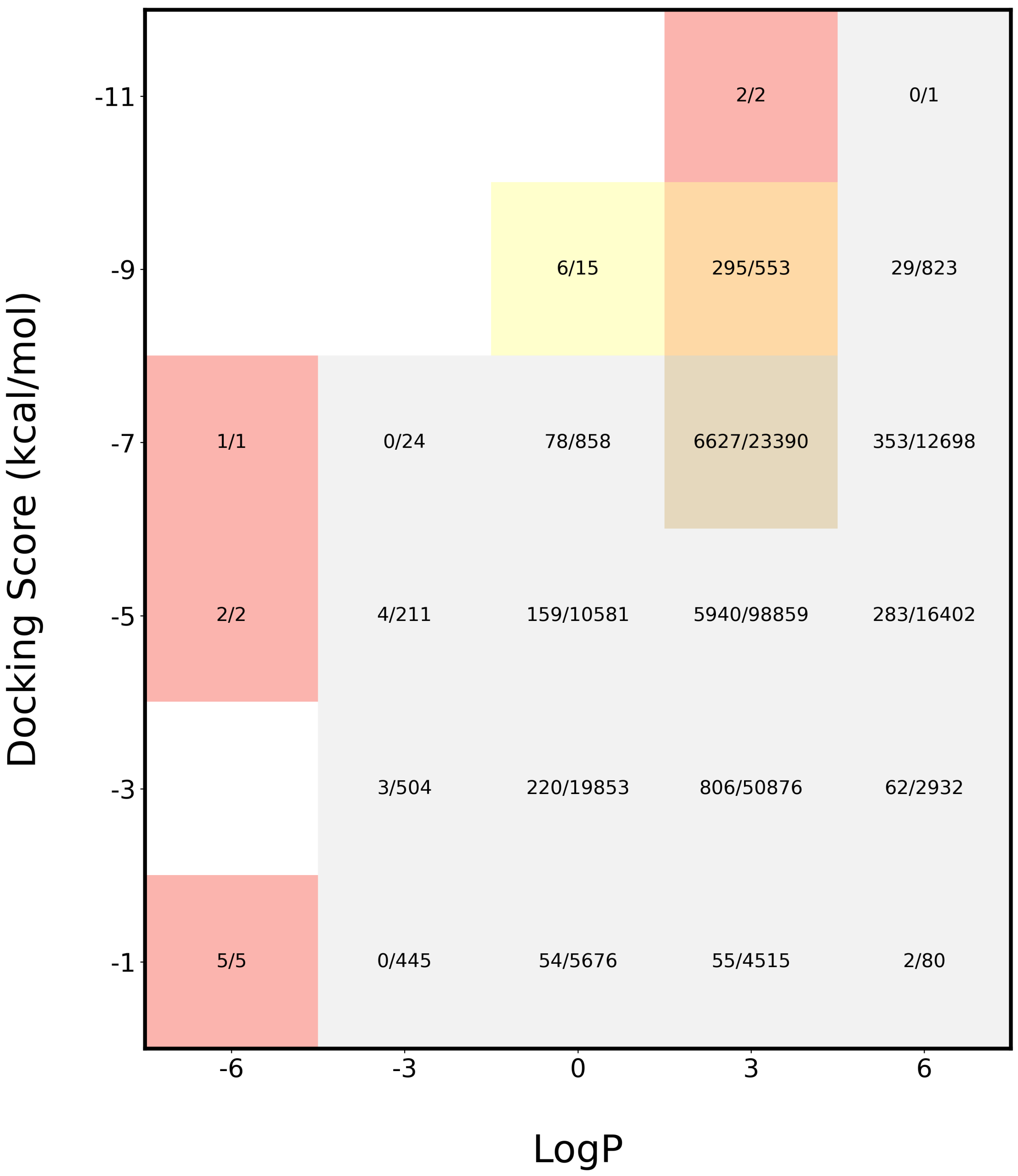

Figure S9. Heatmap of molecules sampled by Deep MO-MEMES using CDDD embeddings out of total molecules in each bucket with LogP between 0 and 5 and high binding affinity to 4BTK

2.3 Deep MO-MEMES with CDDD Embeddings

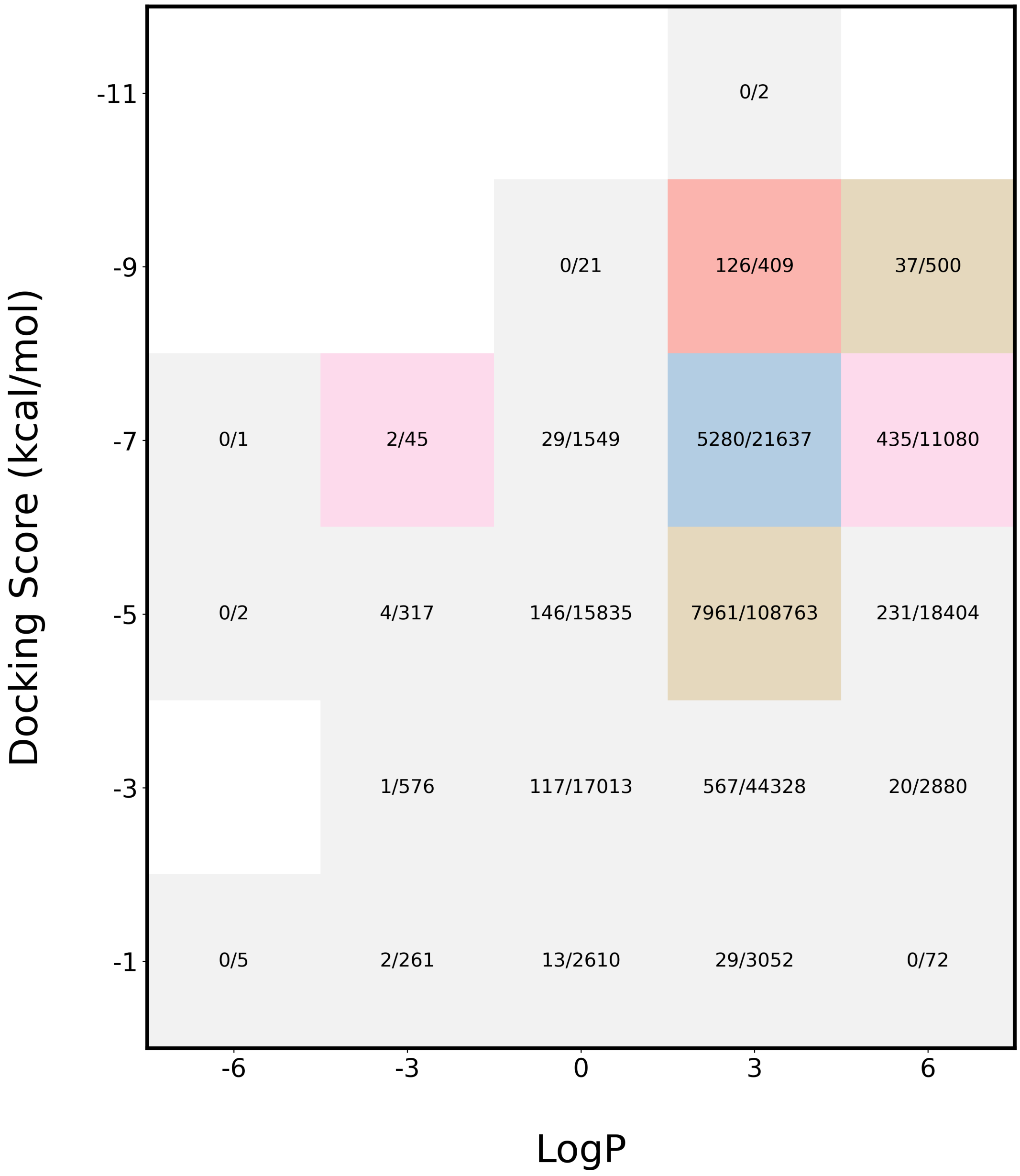

Figure S10. Heatmap of molecules sampled by Deep MO-MEMES using CDDD embeddings out of total molecules in each bucket with LogP between 0 and 5 and high binding affinity to 6LU7

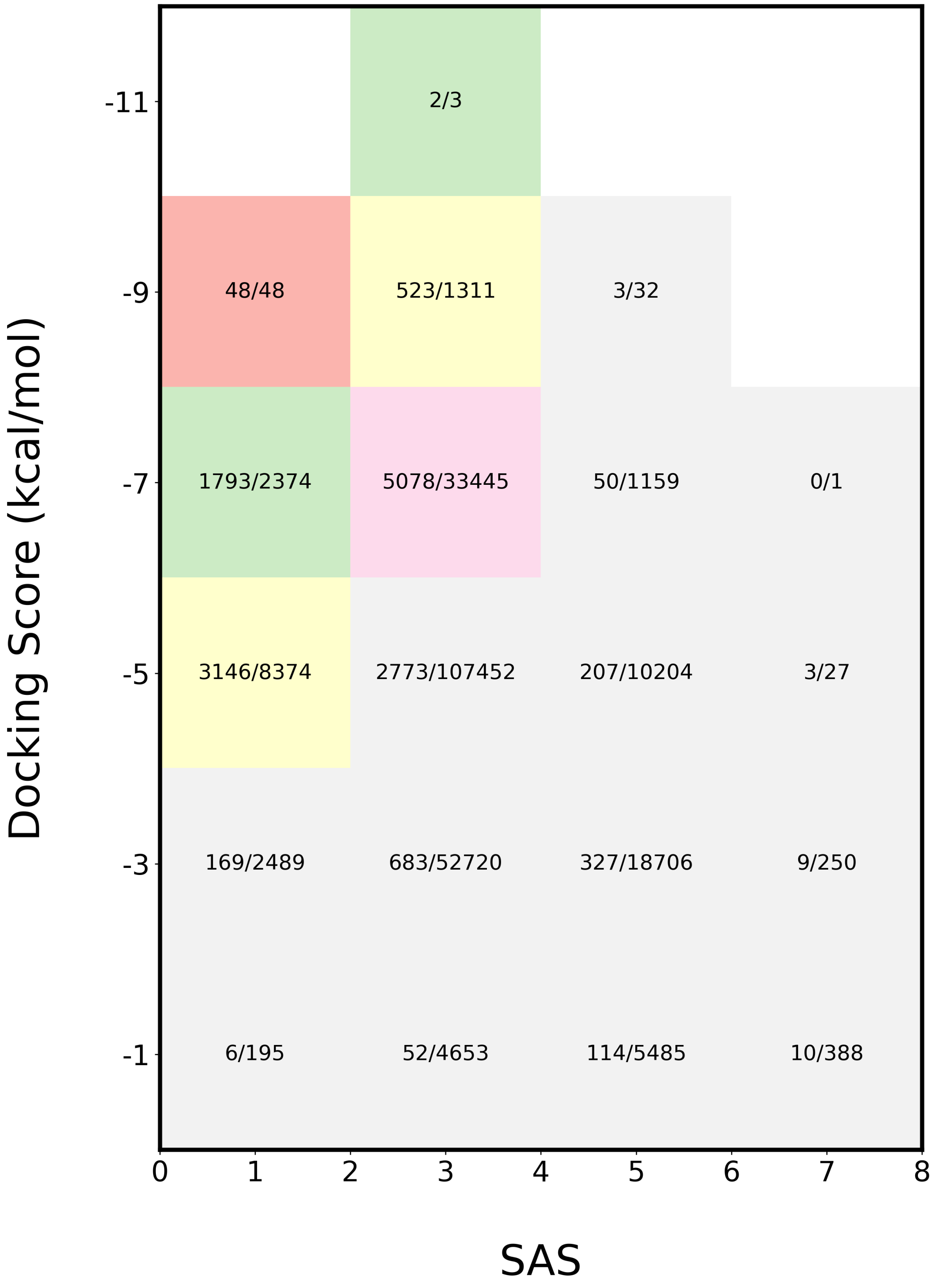

Figure S11. Heatmap of molecules sampled by Deep MO-MEMES using CDDD embeddings out of total molecules in each bucket low SAS and high binding affinity to 4BTK

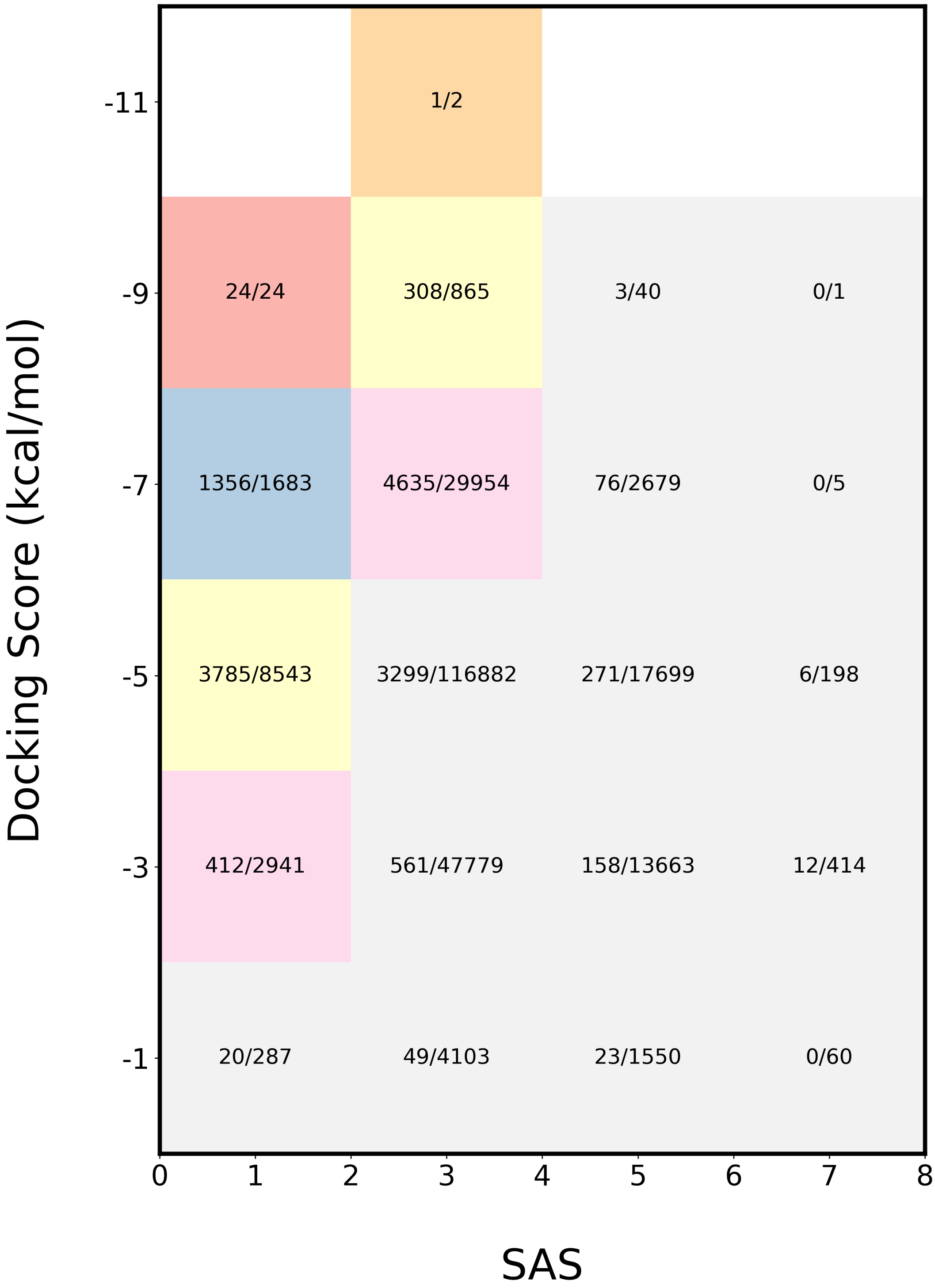

Figure S12. Heatmap of molecules sampled by Deep MO-MEMES using CDDD embeddings out of total molecules in each bucket low SAS and high binding affinity to 6LU7

REFERENCES

Bui, T., Hernández-Lobato, D., Hernandez-Lobato, J., Li, Y., and Turner, R. (2016). Deep gaussian processes for regression using approximate expectation propagation. In *International conference on machine learning* (PMLR), 1472–1481

Dai, Z., Damianou, A., González, J., and Lawrence, N. (2015). Variational auto-encoded deep gaussian processes. *arXiv preprint arXiv:1511.06455*

Damianou, A. and Lawrence, N. D. (2013). Deep gaussian processes. In *Artificial intelligence and statistics* (PMLR), 207–215

Salimbeni, H. and Deisenroth, M. (2017). Doubly stochastic variational inference for deep gaussian processes. *Advances in neural information processing systems* 30
